# Influence of northern limit range on genetic diversity and structure in a widespread North American tree, sugar maple (*Acer saccharum* Marshall)

**DOI:** 10.1002/ece3.3906

**Published:** 2018-02-08

**Authors:** Noémie Graignic, Francine Tremblay, Yves Bergeron

**Affiliations:** ^1^ Institut de Recherche sur les Forêts Université du Québec en Abitibi‐Témiscamingue Rouyn‐Noranda QC Canada

**Keywords:** *Acer saccharum*, northern limit, peripheral populations, population genetics, sugar maple

## Abstract

Due to climate change, the ranges of many North American tree species are expected to shift northward. Sugar maple (*Acer saccharum* Marshall) reaches its northern continuous distributional limit in northeastern North America at the transition between boreal mixed‐wood and temperate deciduous forests. We hypothesized that marginal fragmented northern populations from the boreal mixed wood would have a distinct pattern of genetic structure and diversity. We analyzed variation at 18 microsatellite loci from 23 populations distributed along three latitudinal transects (west, central, and east) that encompass the continuous–discontinuous species range. Each transect was divided into two zones, continuous (temperate deciduous) and discontinuous (boreal mixed wood), based on sugar maple stand abundance. Respective positive and negative relationships were found between the distance of each population to the northern limit (D_north), and allelic richness (*A*_R_) and population differentiation (*F*_ST_). These relations were tested for each transect separately; the pattern (discontinuous–continuous) remained significant only for the western transect. structure analysis revealed the presence of four clusters. The most northern populations of each transect were assigned to a distinct group. Asymmetrical gene flow occurred from the southern into the four northernmost populations. Southern populations in Québec may have originated from two different postglacial migration routes. No evidence was found to validate the hypothesis that northern populations were remnants of a larger population that had migrated further north of the species range after the retreat of the ice sheet. The northernmost sugar maple populations possibly originated from long‐distance dispersal.

## 1 INTRODUCTION

The last glacial maximum (LGM) occurred about 21,000 years before present (‐year BP) and was the coldest period in recent climatic history (Jackson et al., 2000). At that time, North American species were located south of the Laurentian Ice Sheet. After the ice retreated, postglacial vegetation ranges expanded and contracted repeatedly following climatic oscillations (McLachlan, Clark, & Manos, 2005). Given anticipated climate changes, we foresee the northern range expansion of several species (Iverson, Prasad, Matthews, & Peters, 2008).

This shift in range could be particularly marked in peripheral populations located along their distribution edge (Iverson, Schwartz, & Prasad, 2004). Generally isolated and smaller in size (effective population sizes) than core populations (Vucetich & Waite, 2003), these peripheral populations are expected to have a lower level of genetic diversity and a higher level of genetic differentiation among populations following genetic drift or bottlenecks (Eckert, Samis, & Lougheed, 2008; Waters, Fraser, & Hewitt, 2013). In theory, bottlenecked populations are expected to experience a reduction in allelic richness and a limited decrease in heterozygosity. The consequence of this drift would be a reduction in the number of rare alleles (Nei, Maruyama, & Chakrabordy, 1975). The central–marginal hypothesis predicts that those patterns of genetic diversity and structure are the consequences of ecological marginality in populations found at the periphery of the species range compared to core populations (Eckert et al., 2008). In contrast, “range shift following the last glacial maximum” hypothesis proposes that the patterns of genetic diversity in leading edge (i.e., colonizing front) populations are influenced by past climate‐driven range dynamics rather than by demographic and evolutionary processes that are predicted in the central–marginal model (Hampe & Petit, 2005). One problem in distinguishing the relative importance of these processes is that predicted genetic patterns, such as lower genetic diversity and higher differentiation in northern edge populations, may result either from range shifts or from environmental conditions at the edge of a species range. In addition, wind‐pollinated tree species may be buffered against the effect of fragmentation on the genetic structure of peripheral populations due to long‐distance gene flow that contributes to a decrease in differentiation among populations (Kremer et al., 2012).

In the Northern Hemisphere, northern peripheral populations often exhibit local adaptations to cold stress (cold hardiness and earlier bud set in fall) and can potentially be maladapted to climate warming. On the one hand, lower chilling requirements can result in early bud flush in spring under milder environments and greater susceptibility to spring frost damage (Howe et al., 2003). On the other hand, climate warming could also improve their survival and increase the success of sexual reproduction (Alberto et al., 2013).

Sugar maple (*Acer saccharum* Marshall) is a widespread, abundant and long‐lived (300–400‐year) deciduous northeastern American tree (Godman, Yawney, & Tubbs, 1990). This species is a monecious, dichogamous, wind‐pollinated, and shade‐tolerant broadleaf tree (Gabriel, 1968; Gabriel & Garrett, 1984; Logan, 1965) that forms uneven‐aged stands (Majcen, Richard, & Ménard, 1984). It has major economic value as a source of saw timber and syrup production in Canada and the United States (Godman et al., 1990). Sugar maple reaches its northern range at the transition between the temperate deciduous and boreal mixed‐wood forests (Saucier, Grondin, Robitaille, & Bergeron, 2003). Palynological reconstructions support the idea of a constant presence of sugar maple since its establishment in the boreal mixed‐wood forest (Liu, 1990; Richard, 1993). However, two studies have reported the presence of locally larger populations in the past along the shore of the St. Lawrence River (Jetté & Richard, 1992; Richard, Larouche, & Lortie, 1992). Richard and Grondin (2009) reported the establishment of disjointed sugar maple stands around 8,500 calendar years BP (cal. yr BP) in northern Québec. Like many other meridional tree species in the Northern Hemisphere, sugar maple is predicted to migrate north of its current range with anticipated climate change (Goldblum & Rigg, 2005; Iverson et al., 2008).

Earlier studies of sugar maple genetic variation were conducted with provenance tests. These studies revealed clinal variations in response to temperature and moisture stresses (Kriebel, 1957). Sugar maple provenances also showed adaptation to altitudinal gradients for respiration, photosynthesis, and specific leaf weight (Ledig & Korbobo, 1983). Analysis of neutral markers (allozymes and RAPD) revealed weak or no genetic differentiation among sugar maple populations in Canada (Perry & Knowles, 1989; Young, Warwick, & Merriam, 1993) and across its range (Gunter, Tuskan, Gunderson, & Norby, 2000). On a regional scale in Canada, Young et al. (1993) reported the presence of moderate levels of genetic structure among populations, while Diochon, Rigg, Goldblum, and Polans (2003) and Perry and Knowles (1989) found no structure among populations.

In this study, highly polymorphic microsatellite markers developed for sugar maple (Graignic, Tremblay, & Bergeron, 2013) were used to study the genetic diversity and structure of sugar maple populations. Sugar maple populations were selected along three transects that encompass the continuous–discontinuous species range at the transition between the temperate deciduous and boreal mixed‐wood forests. Sexton, McIntyre, Angert, and Rice (2009) highlighted the importance of the replication of edges to disentangle equilibrium limitations (migration‐drift balance) from demographic nonequilibrium characteristics (population expansion, various scenarios of colonization) of range margins. We hypothesized that marginal fragmented northern populations from the boreal mixed‐wood forest would have (1) a lower level of genetic diversity and higher genetic differentiation than populations from the temperate deciduous forest; (2) lower correlations between genetic and geographic distances would be observed in marginal populations; and (3) asymmetric gene flow would occur from core to edge populations under both models. Replication of the edges makes it possible to determine whether the spatial distribution of genetic diversity in sugar maple populations reflects lasting signatures of postglacial range expansion (“range shift following the last glacial maximum”; Hampe & Petit, 2005), or if they are characteristic of peripheral populations per se (central–marginal hypothesis; Eckert et al., 2008). Graignic, Tremblay, and Bergeron (2016) previously reported the presence of higher genetic diversity and a lower inbreeding coefficient in mature trees than in younger regeneration cohorts in three natural sugar maple populations. Mature tree and sapling cohorts were sampled to explore whether the distribution of genetic diversity was different between cohorts in a larger area.

## 2 MATERIALS AND METHODS

### 2.1 Study area and sampling

**Figure 1:**
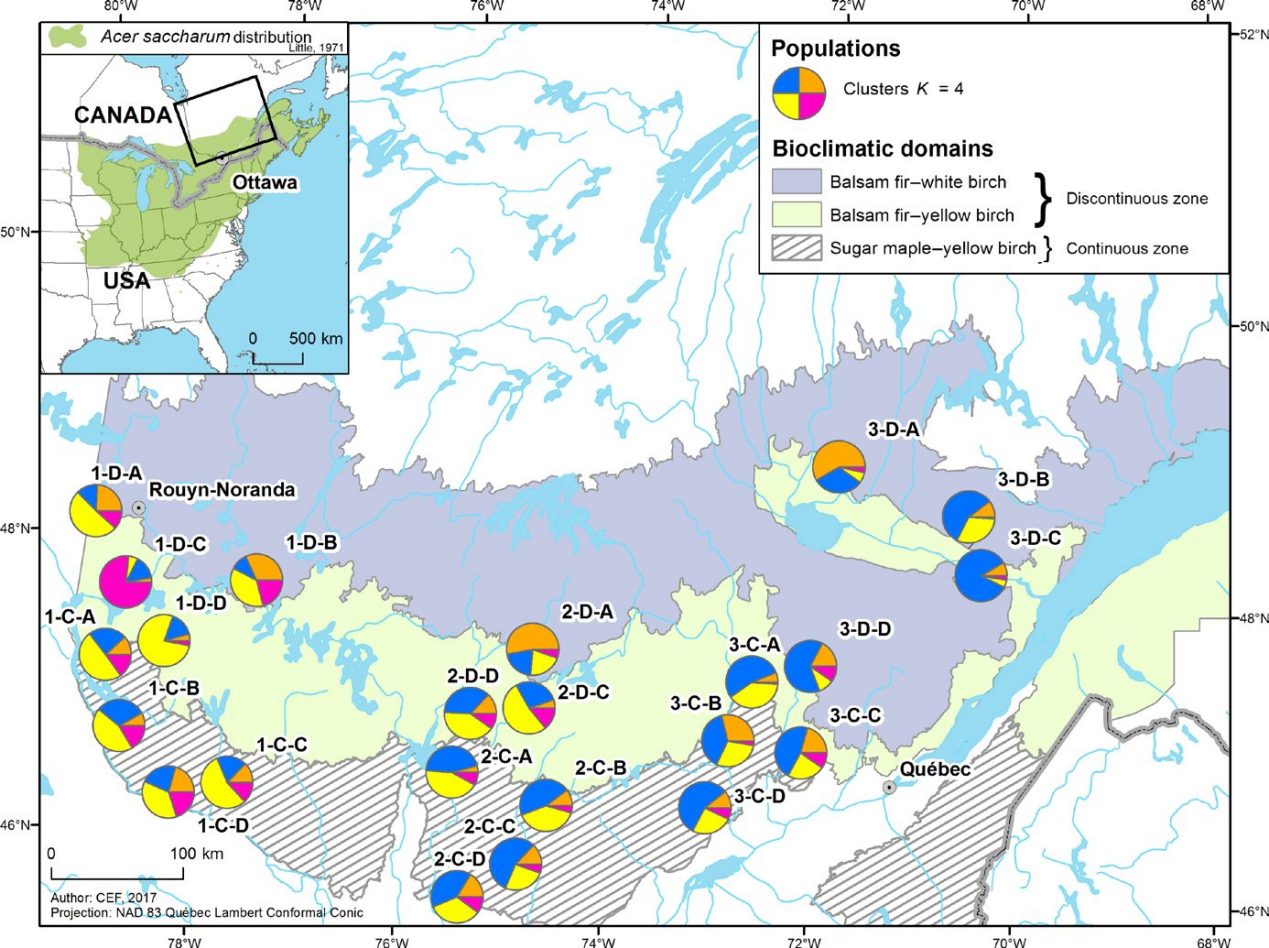
Map of the study area at the northern limit of sugar maple (*Acer saccharum* Marshall) distribution in Québec showing the locations of the 24 study populations (circles; see Graignic et al. (2014) for more details; population 2‐D‐B was thrown out due to amplification errors), and proportional membership of each sugar maple sample in clusters for four genetic groups (*K* = 4) inferred by structure analysis

The study area was located at the northern range limit of sugar maple in Québec (eastern Canada; Figure 1). The presence of sugar maple stands along the latitudinal gradient was estimated from an analysis of large inventory databases (for more details, see Graignic, Tremblay, & Bergeron, 2014). The latitudinal gradient was divided into two zones, based upon the proportion of sugar maple stands in the continuous (temperate deciduous forest) and discontinuous (boreal mixed‐wood forest) zones (referred as C and D zones below, respectively). The continuous zone lies within the sugar maple–yellow birch (*A. saccharum*–*Betula alleghaniensis* Britton) bioclimatic domain where sugar maple is abundant. The discontinuous zone was in the balsam fir–yellow birch (*Abies balsamea* (L.) Miller–*B. alleghaniensis*) bioclimatic domain, and some northern sites were located in the transitional area of the balsam fir–white birch (*A. balsamea*–*Betula papyrifera* Marshall) bioclimatic domain (Saucier et al., 2003; Figure 1).

Sites were distributed along three south–north transects (1, western; 2, center; 3, eastern), with eight sites per transect (four sites per transect per zone; *n *= 24; we analyzed *n* = 23 as population 2‐D‐B needs to be thrown out, see the *Markers genetic diversity* section below for more details; Figure 1). All the sites were old‐growth, uneven‐aged stands, selected to be as similar as possible (for more details, see Graignic et al., 2014). Eleven of the sites selected were either old‐growth or rare forests that are classified as Exceptional Forest Ecosystems (EFE) by the ministère des Forêts, de la Faune et des Parcs du Québec (MFFPQ, Québec Ministry of Forestry, Wildlife and Parks). Data were collected at 24 sites located between 45°51′N and 48°59′N latitude, and 70°21′W and 79°27′W longitude, at elevations ranging between 157 m and 493 m a.s.l.

Tissue samples (usually leaves or bark) were collected from 953 individual sugar maples in 2008 and 2009. As temporal variations in genetic differentiation may be present between different cohorts of regeneration (Foré, Hickey, Guttman, & Vankat, 1992; Mulcahy, 1975), mature trees (≥10 cm diameter at breast height [d.b.h., 1.3 m]; *N* = 12–22) and saplings (1 ≤ d.b.h. <10 cm; *N* = 20) were sampled in each site and analyzed. Samples were frozen (−80°C) or dried (using silica gel) until needed for genetic analyses.

### 2.2 Molecular methods

DNA was extracted using Extract‐N‐Amp™ Plant PCR Kits (Sigma‐Aldrich, Oakville, ON, Canada). All samples were genotyped for 18 variable microsatellite loci using PCR and genotyping protocols as previously described by Graignic et al. (2013). Five different multiplex PCR sets were used, and 37 cycles in the PCR amplification were processed (see Table S1).

### 2.3 Markers genetic diversity

The 2‐D‐B population had to be removed from the analysis due the low number of successful amplification, which was likely caused by poor sample conservation. Accordingly, a total of 913 individuals were selected for the subsequent analysis. For each locus, the total number of alleles (*A*_T_), mean number of alleles (*A*), mean observed (*H*_O_) and expected (*H*_E_) heterozygosity, and inbreeding coefficient (*F*_IS_) were estimated using fstat 2.9.3.2 (Goudet, 2001). Departure from Hardy–Weinberg equilibrium (HWE) per locus in each population and linkage equilibrium between all pairs of loci in each population were calculated using an exact test implemented in genepop 4.2.1 (Rousset, 2008). Markov chain parameters for HWE were 10,000 dememorizations, followed by 500 batches of 5,000 iterations per batch. We corrected for multiple comparisons using a sequential Bonferroni adjustment of *p*‐values to a predetermined experiment‐wise error rate of .05 (Rice, 1989). Null allele frequencies were estimated using freena (10,000 replicates; Chapuis & Estoup, 2007). This software was chosen because it uses the Dempster, Laird, and Rubin (1977) algorithm, which provided the most accurate estimate of several algorithms tested in Chapuis and Estoup (2007). The presence of null alleles overestimated fixation index (*F*_ST_) and genetic distances (Chapuis & Estoup, 2007). We performed a Mantel test (1,000 permutations; using the *mantel* function in the vegan library from the software R 2.13.1; Oksanen et al., 2011; R Development Core Team 2011) between pairwise *F*_ST_ values, with and without correction for null alleles.

### 2.4 Genetic diversity and differentiation between populations and groups

The mean number of alleles per locus (*A*), mean allelic richness (*A*_R_), *H*_O_, *H*_E_, pairwise *F*_ST_, mean pairwise *F*_ST_, and *F*_IS_ were estimated using fstat. The *A*_*R*_ was calculated using rarefaction method, based upon the size of the smallest number of samples. The indices were estimated for each population and on mature trees and saplings for each population separately. To assess whether population diversity parameters differed between zones and transects, *A*_R_, *H*_O_, *H*_E_, *F*_ST_, and *F*_IS_ were calculated by population (all individuals pooled, mature trees, and saplings) within each zone (discontinuous and continuous), transects (1, 2, and 3), and zone in each transect separately using fstat (tested for significance using 1,000 permutations).

### 2.5 Quantitative relationship

Data were analyzed using version 2.13.1 of R (R Development Core Team 2011). Genetic indices *(A*_R_, *H*_O_, *H*_E_ and *F*_IS_) were compared to different characteristics listed below, using a linear mixed‐model analysis (LMM, using the *lme* function in the nlme library; Pinheiro, Bates, Debroy, Sarkar, & Team, 2011) and a linear model for *F*_ST_. Random effects included microsatellite markers for LMM. Different models were run to test the effects of: (1) stand characteristics, such as basal area of mature sugar maple or/and sugar maple sapling (m^2^/ha), density of mature sugar maple or/and sugar maple saplings (stems/ha), population size of mature sugar maple or/and sugar maple saplings (stems), (2) distance of each population to the northern limit (km; see below), and (3) global models with a combination of one model of stand characteristics and the northern limit model. Stand characteristics are included in the analysis because in the central–marginal model, demographic processes are predicted to influence the patterns of genetic diversity. Basal area and density were calculated in a 0.16 ha quadrat on each site (see Graignic et al., 2014). The population size of sugar maple was calculated separately for mature tree and sapling using their densities within the quadrat and stand surface areas. For stand surface areas, we used data from EFE and ecoforestry maps that were obtained from MFFPQ (MFFPQ 2016a,b). Both central–marginal and range shift models predicted that the closer the populations are to the northern limit, the more they are differentiated and the less they are diversified. We fixed the northern limit between balsam fir–white birch (*A. balsamea*–*B. papyrifera*) and balsam fir–yellow birch (*A. balsamea*–*B. alleghaniensis*) bioclimatic domains, and the distance of each sampled population to this limit was estimated with arcmap™ 10.0 (Environmental Systems Research Institute, ESRI, CA, USA); negative values were assigned to sites located to the north of the northern limit, that is, in the balsam fir–white birch (*A. balsamea*–*B. papyrifera*) bioclimatic domain. Correlations between variables were examined, and assumptions of normality and homoscedasticity were graphically tested on global models. Where needed, the data were square‐root‐transformed to meet assumptions. Fits for the 19 models were compared using the Akaike information criterion (AIC; *aictab* in the AIC_c_modavg library; Mazerolle, 2011), corrected for small sample sizes (AIC_C_). We computed AIC_C_ and ΔAIC_C_ weights (ω) to determine the strength of evidence for each model (Burnham & Anderson, 2004). We then performed multimodal inference (*modavg* function in AIC_c_modavg library), where required (ΔAIC_C_ ≤ 4), to calculate the parameter estimates and unconditional 95% confidence intervals (CI).

### 2.6 Bottleneck tests

Evidence of bottlenecks in each population was evaluated using three tests: heterozygote excess, allele frequency mode shift, and *M*‐ratio. For the first test, heterozygote excess, we used bottleneck 1.2.02 (Cornuet & Luikart, 1996) and each population was tested under three mutation models, namely IAM (infinite allele model), SMM (stepwise‐mutation model), and TPM (two‐phase mutational model; Piry, Luikart, & Cornuet, 1999). The significance of the test under all models was performed using one‐tailed Wilcoxon signed‐rank test with 1,000 permutations. The allele frequency mode shift was also conducted in each population using bottleneck (Luikart & Cornuet, 1998).

We also calculated the *M*‐ratio, that is, the mean ratio of the number of alleles (*k*) to the range in allele size (*r*), using m_p_val software (Garza & Williamson, 2001). This method assumes that rare alleles are lost faster during a bottleneck, reducing the number of observed allelic states (*k*) faster than the size range of those alleles (*r*), thereby resulting in a reduced *M*‐ratio (*M* = *k*/*r*). To determine the significance of a ratio, we compared our observed value to critical values (*M*_c_) that were established using the critical_m program (Garza & Williamson, 2001). When the observed *M*‐value was significantly (*p* < .05) below *M*_c_, we assumed that a population had experienced a significant reduction in size (Garza & Williamson, 2001). To calculate *M*_c_ and *p*‐values, we used three different ancestral theta values (θ = 4*N*_e_μ = 1, 5 and 10; *N*_e_, the effective population size; the mutation rate, μ) to account for a wide range of effective population sizes; and the parameters were set as recommended by Garza and Williamson (2001). Each set of simulations consisted of 10,000 iterations.

### 2.7 Isolation by distance

Isolation by distance was investigated using a Mantel test (1,000 permutations; Pearson's correlation; using the *mantel* function in the vegan library in R) between the genetic distance [*F*_ST_/(1 − *F*_ST_)] or [*D*_S_/(1 − *D*_S_)] and geographic distance (km). Pairwise *F*_ST_ and Nei's genetic distance, *D*_S_ (Nei, 1972), were calculated, respectively, using fstat and populations 1.2.32 (Langella, 1999). Geographic distances were estimated using the *earth.dist* function in the fossil library in R (Vavrek, 2012). We performed isolation‐by‐distance analysis for all populations, populations for each zone, and each transect separately.

### 2.8 Genetic structure

Neighbor‐joining (NJ) trees were constructed based on *D*_S_ and using 1,000 bootstraps over loci in populations, one tree for all populations and one tree using cohort separate populations in two parts. NJ trees are useful for describing genetic relationships among populations. The tree was visualized using treeview (Page, 1996).

We used the Bayesian clustering method, implemented in structure 2.3.4 (Pritchard, Stephens, & Donnelly, 2000), to assess the number of populations (*K*), assign individuals to each *K* cluster, detect the population structure, and show whether geographic populations are assigned in genetic populations. Our analyses were based upon an admixture ancestral model with correlated allele frequencies, and a priori sampling locations were used as prior information (LOCPRIOR setting). LOCPRIOR was used to detect any further structure unidentified by standard settings (Hubisz, Falush, Stephens, & Pritchard, 2009). Twenty independent runs were performed for each value of *K* (1–26) with a burn‐in of 100,000 followed by 200,000 MCMC iterations. The most likely value of *K* was determined using the ∆*K* criterion (Evanno, Regnaut, & Goudet, 2005). structure harvester version 0.6.93 was used to extract the results and created a graphic of the ∆*K* criterion (Earl & Vonholdt, 2012). The results were visualized for the best *K*, with distruct version 1.1. (Rosenberg, 2004).

Genetic variation was examined using the hierarchical analysis of molecular variance (AMOVA) based on Φ_PT_ statistic and conducted in genalex 6.5b3 (Peakall & Smouse, 2006). We examined the significant difference (9999 permutations) between transects (1, 2, and 3), between populations within transect and within populations, and between zones (C and D), between populations within zone and within populations for all populations, and for each transect separately. The results of *K*‐means clustering were used to cluster populations into four groups: 1 (1‐D‐A, 1‐D‐B, 2‐D‐A, 3‐D‐A), 2 (1‐D‐D, 1‐C‐A, 1‐C‐B, 1‐C‐C, 1‐C‐D, 1‐D‐C), 3 (2‐D‐C, 2‐D‐D, 2‐C‐A, 2‐C‐B, 2‐C‐C, 2‐C‐D), and 4 (3‐D‐B, 3‐D‐C, 3‐D‐D, 3‐C‐A, 3‐C‐B, 3‐C‐C, 3‐C‐D); and the level of differentiation between groups, between populations within the group, and within populations were tested with AMOVA.

### 2.9 Gene flow and population sizes

We used the Bayesian approach implemented in migrate‐n version 3.6 (Beerli, 2006) to assess the direction and amount of gene flow among populations. We used this software to calculate the posterior probability distribution of the mutation‐scaled effective population size (θ* *= 4*N*_e_μ, where *N*_e_ = effective population size and μ = mutation rate per generation per locus) and the mutation‐scaled past immigration rates (*M* = *m⁄*μ, where *m* = migration rate) using a Metropolis–Hastings algorithm to explore all possible genealogies. The mutation‐scaled population size (θ) for each population and the mutation‐scaled immigration rates (*M*) between each pair of populations were estimated using the Brownian motion mutation model for microsatellites. A coalescent simulation explored the likelihood space for θ and *M*. To limit the number of parameters, the amount of gene flow was estimated between four groups of populations from the transect 1 (1‐D‐C, 1‐D‐D, 1‐C‐A, 1‐C‐B, 1‐C‐C, 1‐C‐D), 2 (2‐D‐C, 2‐D‐D, 2‐C‐A, 2‐C‐B, 2‐C‐C, 2‐C‐D), 3 (3‐D‐B, 3‐D‐C, 3‐D‐D, 3‐C‐A, 3‐C‐B, 3‐C‐C, 3‐C‐D), and the most northern populations (1‐D‐A, 1‐D‐B, 2‐D‐A, 3‐D‐A). The starting values of θ and *M* were generated from the *F*_ST_ estimates, as well as an exponential window prior set for both parameters (min = 0, mean = 5, max = 50 and ∆ = 5 for θ; min = 0, mean = 5, max = 50 and ∆ = 5 for *M*). We replicated two long chains of 10,000,000 genealogies, which were recorded every 10 steps, after a burn‐in period of 10,000. The static heating scheme was set to four chains with temperatures of 100,000.0, 3.0, 1.5, and 1.0, and the swapping interval set at one. The program was run several times adding more sampling schemes and replications. The starting parameters and the resulting estimates were compared until the results were congruent.

## 3 RESULTS

### 3.1 Nuclear microsatellite diversity statistics

The total and mean number of alleles per locus ranged between 4 (SM60) and 31 (SM21A) and from 2.8 (SM60) to 14.8 (SM21A), respectively (Table S1). Significant departure from HWE was observed in 37 (9%) of the 414 possible population–locus combinations (Table S2). Four of the 18 loci (SM22, SM27, SM47, and SM56) failed to meet HWE in ≥6 (26%) populations. For those loci, deviations from HWE were due to heterozygote deficiencies (*F*_IS_ ≥ 0.227; Table S1). Significant linkage disequilibrium between pairs of loci (within populations) was detected in only one of 3,519 tests after Bonferroni correction (SM14 × SM34 in population 3‐C‐A). The loci were therefore all considered genetically independent. Markers that showed a departure from HWE also had high frequencies of null alleles (≥0.10) in most populations (Table S3). Pairwise *F*_ST_ estimates obtained before and after corrections for null alleles were similar (*r* = .98, *p* < .001). Consequently, all loci (18) were retained in the analysis.

### 3.2 Genetic diversity and differentiation

Population 1‐D‐C (Rémigny) from the discontinuous zone of transect 1 had the lowest mean number of alleles per locus (*A*), mean allelic richness (*A*_R_), mean expected heterozygosity (*H*_E_), and inbreeding coefficient (*F*_IS_) for all individuals and both cohorts (mature trees and saplings; Tables 1 and S4). This population had the highest pairwise and global population differentiation (*F*_ST_) for saplings, mature trees, and all individuals pooled (Tables 1, and S4, S5). Pairwise *F*_ST_ was significant between 176, 73, and 51 pairs on 253 pairs for the individuals pooled, saplings, and mature trees, respectively, after adjustment for multiple comparisons (Table S5). The range of *A*,* A*_R_, *H*_E_, *F*_IS_, and *F*_ST_ for sugar maple population with all individuals pooled was 6.6–9.9, 5.8–7.6, 0.637–0.715, −0.051–0.302, and 0.009–0.041, respectively (Table 1).

**Table 1:** Genetic variability estimates of sugar maple (*Acer saccharum*) populations in Québec for all individuals

Population ID	Population name	*N*	*A*	*A*_R_	*H*_O_	*H*_E_	*F*_IS_	*F*_ST_
1‐D‐A	Lac Labelle	40	7.8	6.6	0.532	0.684	0.222	0.020
1‐D‐B	Lac Okiwakamik	40	8.1	7.0	0.590	0.693	0.149	0.015
1‐D‐C	Rémigny	32	6.6	5.8	0.670	0.637	−0.051	0.041
1‐D‐D	Lac de la Tour	40	7.9	6.9	0.608	0.700	0.131	0.017
1‐C‐A	Lac St Amand	40	8.7	7.5	0.620	0.704	0.120	0.011
1‐C‐B	Kipawa	39	8.0	7.2	0.697	0.702	0.007	0.013
1‐C‐C	Lac Six Milles	40	8.6	7.4	0.664	0.698	0.049	0.018
1‐C‐D	Lac Percival	40	8.5	7.3	0.546	0.696	0.216	0.011
2‐D‐A	Lac Pénobscot	40	8.1	7.1	0.623	0.709	0.121	0.019
2‐D‐C	Réservoir Mitchinamécus	40	8.5	7.4	0.548	0.715	0.234	0.014
2‐D‐D	Lac des Polonais	40	8.6	7.5	0.516	0.684	0.246	0.011
2‐C‐A	Montagne du Diable	40	8.2	7.1	0.596	0.684	0.129	0.012
2‐C‐B	Lac Ecuyer	40	8.6	7.3	0.505	0.698	0.276	0.012
2‐C‐C	Lac Marie‐Lefranc	42	8.6	7.2	0.589	0.691	0.147	0.010
2‐C‐D	Lac de l'Ecluse	40	8.6	7.2	0.569	0.687	0.172	0.009
3‐D‐A	Lac Patrick	40	8.0	7.0	0.496	0.710	0.302	0.021
3‐D‐B	Fjord du Saguenay	40	7.8	7.0	0.681	0.687	0.008	0.017
3‐D‐C	Baie Eternité	40	7.4	6.3	0.639	0.661	0.033	0.026
3‐D‐D	Lac Edouard	40	8.3	6.5	0.656	0.694	0.054	0.019
3‐C‐A	Lac Paul	40	8.4	7.1	0.548	0.699	0.215	0.019
3‐C‐B	Lac Dickey	40	9.0	7.6	0.618	0.700	0.117	0.011
3‐C‐C	Lac Grandbois	40	8.0	6.9	0.499	0.691	0.278	0.012
3‐C‐D	Lac Larose	40	8.2	7.0	0.716	0.715	−0.000	0.012
Means		40	8.2	7.0	0.597	0.693	0.138	0.016

*N*, sample size; *A*, mean number of alleles; *A*_R_, mean allelic richness; *H*_O_, mean observed heterozygosity; *H*_E_, mean expected heterozygosity; *F*_IS_, inbreeding coefficient; *F*_ST_, mean pairwise *F*_ST_.

Test groups showed that mean *A*_R_ was significantly lower in populations from the discontinuous vs. the continuous zone (6.8 vs. 7.2; Tables 2 and S6). This difference in mean *A*_R_ between zones was significant only in transect 1 for the individuals pooled (6.6 vs. 7.3; Table 2), mature trees (5.1 vs. 5.8; Table S6), and saplings (5.1 vs. 5.5; Table S6). The mean *F*_ST_ was higher in populations from the discontinuous vs. the continuous zone (0.025 vs. 0.009) and in transect 1 (0.030 vs. 0.004) but not transects 2 and 3 (Table 2). Population differentiation (*F*_ST_) was different between discontinuous and continuous zones both for mature trees (0.034 vs. 0.007) and saplings (0.029 vs. 0.003) in transect 1 (Table S6). No significant differences for *A*_R_ and *F*_ST_ were found between transects (Table 2), between zones for transect 2 and 3 (Table 2), and between cohorts (mature trees and saplings; Table S7), or for all other genetic parameters.

**Table 2:** Comparison of mean genetic variability estimates between populations of zones, transects, and zones in each transect of sugar maple (*Acer saccharum*) in Québec for all individuals

Genetic indices	Zones			Transects												
	D	C	*p*‐Value	1	2	3	*P*‐value	1 Zone			2 Zone			3 Zone		
								D	C	*p*‐Value	D	C	*p*‐Value	D	C	*p*‐Value
*A*_R_	6.840	7.246	**0.0080**	6.970	7.272	6.941	0.2450	6.601	7.339	**.0100**	7.338	7.222	.7380	6.707	7.176	.0880
*H*_O_	0.600	0.600	0.9950	0.616	0.567	0.611	0.2610	0.597	0.634	.4460	0.566	0.567	.9870	0.623	0.599	.5800
*H*_E_	0.689	0.698	0.2110	0.691	0.695	0.694	0.8610	0.680	0.701	.0780	0.703	0.690	.2630	0.687	0.701	.1960
*F*_IS_	0.129	0.140	0.7730	0.108	0.184	0.120	0.2520	0.122	0.095	.6760	0.194	0.178	.8380	0.093	0.146	.4230
*F*_ST_	0.025	0.009	**0.0010**	0.017	0.009	0.016	0.4630	0.030	0.004	**.0050**	0.014	0.005	.3910	0.022	0.011	.2490

*A*_R_, mean allelic richness; *H*_O_, mean observed heterozygosity; *H*_E_, mean expected heterozygosity; *F*_ST_, mean pairwise *F*_ST_; *F*_IS_, inbreeding coefficient; D, discontinuous zone; C, continuous zone. Significant values (α = 0.05) given in bold.

### 3.3 Quantitative relationship

**Figure 2:**
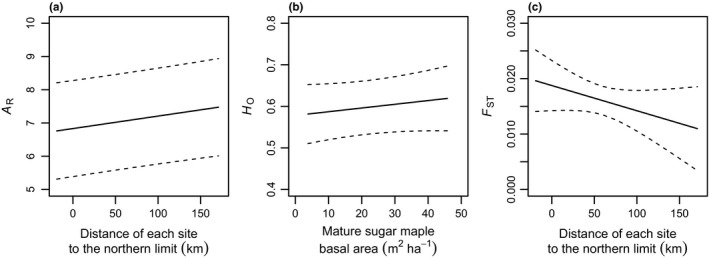
Predicted sugar maple (*Acer saccharum*) genetic indices (*A*_R_, *H*_O_ and *F*_ST_) in response to all influential explanatory variables in best supported models based on multimodel averaging of all candidate models (*n* = 414 for *A*_R_ and *H*_O_, and *n* = 23 for *F*_ST_) in Québec. Dashed lines indicate 95% confidence intervals. *A*_R_, allelic richness; *H*_O_, observed heterozygosity

There were 10, 7, 19, 11, and 10 best models (∆AIC_C_ ≤ 4.0; Table S8) for *A*_R_, *H*_O_, *H*_E_, *F*_IS_, and *F*_ST_, respectively. Parameter estimates derived from multimodel inference show no influence on *H*_E_ and *F*_IS_ for all variables tested, that is, the distance to the northern limit variable and the six stand characteristics variables (Table 3). A positive relationship and negative relationship were found between the distance of each population to the northern limit (D_north), and *A*_R_ and *F*_ST_, respectively. A positive relationship between mature sugar maple basal area and *H*_O_ was also observed (Table 3, Figure 2).

**Table 3:** Parameter estimates and unconditional confidence intervals from multimodel inference of sugar maple (*Acer saccharum*) *A*_R_, *H*_O_, *H*_E_ (√data), *F*_IS_, and *F*_ST_ in Québec

Explained variables	Explanatory variables	Model‐averaged estimate	Unconditional *SE*	95% Confidence interval	
				Lower	Upper
*A*_R_	m_ers_BA	0.00605	0.00569	−0.0051	0.01721
	s_ers_BA	0.00155	0.0194	−0.03647	0.03957
	m_ers_d	0.0006	0.00048	−0.00034	0.00155
	s_ers_d	0.00002	0.00005	−0.00007	0.00011
	m_ers_PS	0	0	−0.00005	0
	s_ers_PS	0	0	0	0
	D_north*	0.00378	0.00119	0.00146	0.00611
*H*_O_	m_ers_BA*	0.00154	0.00062	0.00031	0.00276
	s_ers_BA	−0.00438	0.0023	−0.00889	0.00013
	m_ers_d	0.00011	0.00006	0	0.00022
	m_ers_PS	0	0	0	0
	s_ers_PS	0	0	0	0
	D_north	0.00011	0.00019	−0.00027	0.00049
*H*_E_	m_ers_BA	0.0002	0.0002	−0.0002	0.00059
	s_ers_BA	−0.00051	0.00076	−0.002	0.00097
	m_ers_d	0.00002	0.00002	−0.00002	0.00006
	s_ers_d	0	0	0	0
	m_ers_PS	0	0	0	0
	s_ers_PS	0	0	0	0
	D_north	0.00004	0.00004	−0.00004	0.00013
*F*_IS_	m_ers_BA	−0.00171	0.00094	−0.00355	0.00012
	s_ers_BA	0.00566	0.00347	−0.00115	0.01247
	m_ers_d	−0.0001	0.00008	−0.00026	0.00006
	s_ers_d	0	0.00001	−0.00002	0.00001
	m_ers_PS	0	0	0	0
	s_ers_PS	0	0	0	0
	D_north	−0.00003	0.00022	−0.00046	0.00041
*F*_ST_	m_ers_BA	−0.0002	0.00015	−0.00049	0.0001
	s_ers_BA	−0.00037	0.00052	−0.00139	0.00064
	m_ers_d	−0.00002	0.00001	−0.00004	0.00001
	s_ers_d	0	0	0	0
	m_ers_PS	0	0	0	0
	s_ers_PS	0	0	0	0
	D_north*	−0.00006	0.00003	−0.00011	−0.00001

Asterisks identify parameters where confidence intervals excluded 0.

*A*_R_, mean allelic richness; *H*_O_, mean observed heterozygosity; *H*_E_, mean expected heterozygosity; *F*_ST_, mean pairwise *F*_ST_; *F*_IS_, inbreeding coefficient.

Term abbreviations: m_ers_BA, mature sugar maple basal area (m^2^/ha); s_ers_BA, sugar maple sapling basal area (m^2^/ha); m_ers_d, mature sugar maple density (stems/ha); s_ers_d, sugar maple sapling density (stems/ha); m_ers_PS, mature sugar maple population size (stems); s_ers_PS, sugar maple sapling population size (stems); D_north, distance of each site to the northern limit (km); *SE*, standard error.

### 3.4 Bottlenecks

Inferences to be drawn from the heterozygosity excess tests were influenced by the mutational model. Under the IAM, most of the populations were in excess of heterozygotes except for populations 1‐C‐D, 2‐C‐D, and 2‐D‐D (Table S9). The mode shift model, TPM, and SMM showed no population in bottleneck. For the *M*‐ratio, no bottleneck was detected, except for three populations (1‐D‐C, 1‐D‐D, and 3‐C‐A) for θ = 1 (Table S9). No significant bottlenecks were found for the other θ values (Table S9).

### 3.5 Isolation by distance

Isolation by distance was observed among populations in Québec (*r* = .36, *p* < .001) and in zones (*r* = .35, *p *= .008 and *r* = .36, *p* = .002, respectively, for continuous and discontinuous zones) using *F*_ST_/(1 − *F*_ST_). Tests were not significant when each transect was tested separately (*r* = −0.06, *p *= .533; *r* = .33, *p* = .139; and *r* = .06, *p* = .390, respectively, for transects 1, 2, and 3). Similar results were obtained using *D*_S_/(1 − *D*_S_) (Table 4).

**Table 4:** Isolation‐by‐distance analysis results performed separately for different geographic groups

Group	Number of populations	*F*_ST_/(1 − *F*_ST_)		*D*_S_/(1 − *D*_S_)	
		*r*	*p*	*r*	*p*
All populations	23	.36	.001	.36	.001
Zone					
Discontinuous	11	.35	.008	.36	.005
Continuous	12	.36	.002	.36	.002
Transect					
1	8	−.06	.533	−.05	.532
2	7	.33	.139	.26	.184
3	8	.06	.390	.02	.476

*F*_ST_, genetic differentiation among populations; *D*_S,_ genetic distance.

### 3.6 Genetic structure

The neighbor‐joining tree separated the populations of the transect 1 (except for 1‐D‐C, Rémigny) from the other two transects (Figure S1). However, bootstrap support (<25%) was poor overall. The strongest link was found between populations 2‐D‐A and 3‐D‐A with 79% bootstrap support. When the populations were divided into two groups (mature trees and saplings), similar groupings occurred for 12 populations (bootstrap support range: 20–99), of which seven had bootstrap support ≥50% (Figure S1).

The structure output produced the highest value of ∆*K* at *K* = 4 (Figure S2). Most individuals had partial membership in multiple clusters (orange, blue, yellow, and pink clusters; Figure 1) indicating high levels of admixture; however, a structure could still be drawn. Individuals from the northernmost populations in 1‐D‐A, 1‐D‐B, 2‐D‐A, and 3‐D‐A were predominantly assigned to the orange cluster. The eastern and western transects were largely admixed with higher average proportions of membership to the blue cluster in the former and to the yellow cluster in the latter. High assignment probabilities to the blue and yellow clusters were found in the central transect (2). Individuals from population 1‐D‐C (Remigny) were more homogeneous and associated with the pink cluster.

AMOVA showed no biologically meaningful differentiation among populations at the level of zone (0%) and transect (1%, *p* = .000; Table S10). Most of the genetic variation was found within populations for all groupings (within 97%, *p* = .000; among 2%–3%, *p* = .000; Table S10).

### 3.7 Gene flow and population sizes

The largest mean scaled effective population size (θ) was in the eastern area of Québec (transect 3; mean θ = 3.68), and the smallest was in the center (transect 2; mean θ* *= 1.22; Table 5, Figure S3). Northern populations also had a low mean scaled effective population size (θ* *= 1.43). Remarkable levels of gene flow were observed among populations from the three transects, with a slightly more scaled immigration rate (*M*) from the east (transect 3) than from the other two transects (Table 5, Figure S3). Gene flow was asymmetrical, with higher migration rates from the south (transects 2 and 3) toward the northern populations, while low and equal migration rates were detected between the west (transect 1) and northern populations (Table 5, Figure S3).

**Table 5:** Mean mutation‐scaled population size (θ* *= 4*N*_e_μ, where *N*_e_ = effective population size and μ = mutation rate per generation per locus) and mean mutation‐scaled immigration rate (*M* = *m*/μ, where *m* = migration rate) between pairs of sugar maple (*Acer saccharum*) populations. Both parameters are estimated using migrate‐n (Beerli, 2006)

	θ	NP	ST1	ST2	ST3
NP→*i*	1.43 (0.57–2.27)	—	7.8 (6.7–8.8)	8.5 (6.7–9.9)	7.6 (5.8–9.8)
ST1→*i*	2.77 (1.7–3.80)	6.6 (4.6–7.5)	—	15.5 (14.6–16.4)	15.6 (14.5–16.9)
ST2→*i*	1.22 (0.03–2.37)	16.7 (15.5–17.9)	17.8 (16.7–18.8)	—	15.0 (13.9–16.1)
ST3→*i*	3.68 (2.33–5.30)	25.7 (24.8–26.7)	20.8 (19.1–22.5)	16.9 (15.6–18.8)	—

95% confidence intervals are shown in parentheses. Source groups are presented in the first column, and sink groups are presented in the first row. NP, 1‐D‐A, 1‐D‐B, 2‐D‐A, 3‐D‐A; ST1, 1‐D‐C, 1‐D‐D, 1‐C‐A, 1‐C‐B, 1‐C‐C, 1‐C‐D; ST2, 2‐D‐C, 2‐D‐D, 2‐C‐A, 2‐C‐B, 2‐C‐C, 2‐C‐D; and ST3, 3‐D‐B, 3‐D‐C, 3‐D‐D, 3‐C‐A, 3‐C‐B, 3‐C‐C, 3‐C‐D.

## 4 DISCUSSION

Range limits in trees are associated with demographic and ecological factors that result in population isolation and fragmentation at landscape level. How fragmentation affects evolutionary processes is unlikely to be universal and to depend upon several factors, such as the effective population size, the extent of gene flow, and the level of connectivity between populations at the range edge. This is the first time that the genetic diversity and structure of sugar maple populations have been studied over a wide range at its northern range. Our results showed evidence of the impact of marginality on sugar maple genetic structure. Lower allelic richness (*A*_R_) and higher genetic differentiation (*F*_ST_) between populations were detected in the discontinuous zone where populations are at the periphery of the sugar maple range in Québec. Furthermore, gene flow between zones was asymmetric, mostly directed outward from the populations of the temperate deciduous forest. A high level of admixture and a weak population structure were also detected. Most genetic variation was found within sugar maple populations, a frequent pattern for long‐lived perennials, outcrossing, and wind‐dispersed seeds plants (Nybom, 2004). Higher level of gene flow occurred from the southern into the four northernmost populations. This asymmetrical gene flow may contribute to increasing effective population size and genetic variation in marginal populations (Alleaume‐Benharira, Pen, & Ronce, 2006). It may also introduce alleles preadapted to a warmer climate (nonlocal genes; Kremer et al., 2012), which may facilitate adaptation in the boreal mixed‐wood sugar maple populations in the context of climate change. A genetic difference between our western transect and others transects led us to think that migration influence this structure, that is the range shift model rather than the ecological marginality in itself was the best explaining model for sugar maple.

### 4.1 Genetic diversity levels in sugar maple populations

The eighteen microsatellite markers developed by Graignic et al. (2013) revealed a high level of genetic diversity in sugar maple populations (see Table 1). Only two studies using microsatellite markers on natural population of sugar maple (Graignic et al., 2016; Khodwekar, Staton, Coggeshall, Carlson, & Gailing, 2015) were available for comparison with our results. Our results were in the range reported by Khodwekar et al. (2015) (*H*_E_ = 0.822), by Graignic et al. (2016) (*H*_E_ = 0.689), and other species such as *Acer mono* Maxim. (*H*_E_ = 0.80, range: 0.70–0.85; Kikuchi, Shibata, Tanaka, Yoshimaru, & Niiyama, 2009; Takayama, Sun, & Stuessy, 2012) and *Acer campestre* L. (*H*_E_ = 0.602, range: 0.509–0.699; Chybicki, Waldon‐Rudzionek, & Meyza, 2014; Table S11).

Large variation was observed in the inbreeding coefficient estimates for sugar maple populations in Québec (*F*_IS_ = 0.138, range: −0.051–0.302). Similar results were reported with microsatellites for *Acer takesimense* (*F*_IS_ = 0.28, range: 0.08–0.47; Takayama, Sun, & Stuessy, 2013) and for *A. campestre* (*F*_IS_ = 0.107, range: 0.015–0.300; Chybicki et al., 2014; Table S11).

### 4.2 Genetic variation and latitudinal gradient

We found a lower allelic richness (*A*_R_) and higher genetic differentiation (*F*_ST_) between populations in the discontinuous zone that represents the periphery of sugar maple range in Québec (Table 2). The relationship between the proximity to the northern limit and genetic diversity was negative, whereas it was positive with genetic differentiation (Table S8, 3, Figure 2a,c). The individuals forming the most northern populations of each transect (1‐D‐A, 1‐D‐B, 2‐D‐A and 3‐D‐A) were assigned to a distinct group with structure (Figures 1 and S3). These results demonstrated the effect of a latitudinal gradient on sugar maple genetic diversity.

However, when these relationships were tested for each transect separately, the pattern (discontinuous/continuous) remained significant only for the western transect (transect 1). The reduction in *A*_R_ and the increase in *F*_st_ along the western transect are most likely the consequence of successive founder events that followed the retreat of the ice sheet and are more consistent with the range shift hypothesis (Hampe & Petit, 2005). These relationships were not due to isolation by distance because no significant correlation between genetic and geographic distances was detected within transect (Table 4). Migration routes could explain the difference between western transect and other transects, see Section CONFLICT OF INTEREST. Effects of historical factors on genetic processes might be enhanced by demographic and ecological conditions. A lower level of sugar maple recruitment (lower seedlings densities) in the discontinuous zone was previously reported in the western transect (Graignic et al., 2014). The association between lower sexual recruitment and lower genetic diversity in peripheral populations was also suggested for *Thuja occidentalis* L. (Paul, Bergeron, & Tremblay, 2014; Xu, Tremblay, Bergeron, Paul, & Chen, 2012) and for the dwarf thistle *Cirsium acaule* (L.) A.A. Weber ex Wigg (Jump, Woodward, & Burke, 2003) at their northern limits. The northwestern region is included within a larger physiographic region that was created by lacustrine deposits from the proglacial lakes Barlow and Ojibway (Veillette, 1994). Clay and organic deposits increase from south to north, while rock and till deposits, which constitute the most favorable habitats for maple establishment, tend to decrease. These conditions contribute to further limiting the distribution of the species (Tremblay, Bergeron, Lalonde, & Mauffette, 2002). Lower sexual regeneration, combined with the reduction in the number of suitable sites for recolonization following disturbances, may have limited the long‐term maintenance of the species in local stands.

In the eastern and central regions, *A*_r_ and *F*_st_ did not change from core to edge populations, indicating no direct effect of fragmentation on genetic diversity of sugar maple populations. Paleoecological records support the constant presence of sugar maple in mixed‐wood or boreal forests since its establishment (Liu, 1990; Richard, 1993). We previously reported constant sugar maple recruitment over time in these northern populations (Graignic et al., 2014). Therefore, it is plausible that the number, size, and connectivity of suitable habitat patches were sufficient to maintain stable levels of gene flow and genetic diversity toward the range edges in populations from the central and eastern transects (Sexton et al., 2009).

No recent bottleneck was detected under the SMM, TPM, and mode shift indicator tests, while most populations were in bottleneck under the IAM (Table S9). Under IAM, microsatellite loci have been shown to exhibit heterozygosity excess in stable populations (Luikart & Cornuet,^1998^). This result suggests there have been no significant reductions in effective population size. The *M*‐ratio analysis proved to be rather sensitive to the choice of θ. The results showed that only three populations may have undergone an historical bottleneck when the mutation‐scaled effective population size was estimated at θ = 1, but no bottleneck was detected at θ = 5 or higher (Table S9). Among these populations, only individuals from population 1‐D‐C (Rémigny) were assigned to a distinct cluster (defined by structure) and had probably undergone a bottleneck in a distant past (Table 5 and Figure S3; see below for the Section 4.3).

Many empirical studies on forest tree species do not support the central–marginal hypothesis (Gapare, Aitken, & Ritland, 2005; González‐Martínez, Gil, & Alía, 2005; Hoban et al., 2010; Jadwiszczak, Banaszek, Jabłońska, & Sozinov, 2011; Rajora, Deverno, Mosseler, & Innes, 1998). For example, the results of Hoban et al. (2010) best supported the range shift model as the determinant of genetic structure for *Juglans cinerea* L. In a recent study, Chybicki et al. (2014) reported lower genetic variation and higher divergence rates in *A. campestre* populations that were located closer to the northern margin of the species range. They interpreted this latitudinal genetic gradient as a result of postglacial recolonization because no relationship was found between *A. campestre* density and genetic structure. Even if only populations residing in the northern part of the species range were sampled when we compare to the whole sugar maple range, we were able to determine that the range shift model rather than the ecological marginality in itself was the best explaining geographical variation in the genetic structure of sugar maple.

### 4.3 Special case of 1‐D‐C, Rémigny

The population from Rémigny (1‐D‐C) had the lowest level of diversity (*A*,* A*_R_, *H*_E_, and *F*_IS_), and the highest *F*_ST_ (Table 1), and a bottleneck was detected (Table S9; *M*‐ratio at θ* *= 1). Both neighbor‐joining trees and structure analysis clearly disjointed this population from all the others (Figure S1, 1). These differences were probably due to the recent establishment of sugar maple at this site. A study conducted by Pilon and Payette (2015) at a nearby sugar maple stand (about 7 km away) dated sugar maple's arrival after a fire, which occurred 160–220 yrs ago. According to Pilon and Payette (2015), the presence of sugar maple at Rémigny was sporadic or scarce during the last millennia. Probably, its presence was too recent and the time lapses too short to reach a level of genetic diversity similar to the other sugar populations. In their work on *Pinus ponderosa*, Lesser, Parchman, and Jackson (2013) reported that more than 230 yrs following stand establishment was required to reach allele saturation.

### 4.4 Mature tree and sapling cohorts

In many coniferous tree species, the level of heterozygosity increases with the age of the trees (seeds vs. adults, seeds vs. seedlings, different age classes), possibly due to higher fitness of heterozygous individuals (Bush & Smouse, 1992; Nijensohn, Schaberg, Hawley, & Dehayes, 2005). Ballal (1994) reported that sugar maple embryos had lower heterozygosity and higher *F*_IS_ than seedlings (1‐yr to ≤1 cm basal area) and mature trees (≥30 cm dbh). Graignic et al. (2016) reported the same difference between younger regeneration cohorts (seedlings and saplings) and mature trees. Foré et al. (1992) found no difference between sugar maple embryos, 1‐yr seedlings, and three other cohorts (dbh ≤2 cm, 15–25 cm and ≥40 cm). We found a significant positive relationship between mature sugar maple basal areas and *H*_O_ (Table S8, 3 and Figure 2), but no relationship with *F*_IS_. Overall, these results did not support the hypothesis of higher heterozygosity in older cohort.

### 4.5 Genetic signature of migration routes

We observed a genetic difference between the eastern and the western transects (blue and yellow clusters, see Figure 1). The largest population size was found in the eastern populations followed by the western and the central populations, respectively (Table 5; Figure S3). This result may possibly represent two distinct migration routes (first from the east, then from the west) that separated a long time ago, or originated from different glacial refugia.

Earlier sugar maple migrations in Québec started about 9,900 cal. yr BP in southeastern Québec (step 1 in Figure S4; Lavoie & Richard, 2000; see all steps in Figure S4), and sugar maple pollen was recorded 6,300 cal. yr BP in the southwest of Québec (step 5; Vincent, 1973). Therefore, the arrival of sugar maple in western Québec occurred around 3,000 yr later than it did in the east, which corroborated evidence for two distinct migration routes in Québec. Our hypothesis also matches Braun's hypothesis, which was based on pollen data analysis that suggests beech–maple associations moved northward via two routes following ice retreat (for more details, see Figure S4; Braun, 1950). Jackson et al. (2000) identified, from pollen and macrofossils data, two maple glacial refugia during the LGM (21,000 cal. yr BP; Figure S4). Interestingly, a genetic signature of these same refugia was identified for red maple (*Acer rubrum* L.; McLachlan et al., 2005) and multiple refugia for sugar maple (Vargas‐Rodriguez, Platt, Urbatsch, & Foltz, 2015).

Southern populations in central Québec (transect 2) had a lower population size (Table 5; Figure S3) and were clustered with western and eastern populations (transects 1 and 3; Figure 1). We hypothesized that these populations originated from an admixture of westward and eastward Québec migrations (step 7 in Figure S4), with greater contributions from the eastward advance (Figure S3). Similar routes for northernmost populations could also be drawn, but with considerably more contributions from south–central–eastern populations (transects 2 and 3; step 7 in Figure S4) because (1) very low contributions from the southwestern populations to the northernmost populations were found (Table 5, Figure S3), and (2) western populations arrived later than did the eastern populations in Québec (Lavoie & Richard, 2000; Vincent, 1973).

## 5 CONCLUSIONS

The inclusion of among‐edge population comparisons provided in this study provides new insights into the question of whether the “ecology marginality at the periphery” (central–marginal hypothesis; Eckert et al., 2008) or “range shift following the last glacial maximum’ (Hampe & Petit, 2005) models best describes the pattern of genetic variation in sugar maple populations. The pattern of variation in genetic diversity along the western transect is best supported by the range shift model. No evidence was found to validate the hypothesis that northern populations were remnants of a larger population that had migrated further north of the species range after retreat of the ice sheet. It is possible that northernmost populations originated from long dispersal distances (Cain, Milligan, & Strand, 2000). Additional paleoecological data would be essential to accurately establish the arrival of the northernmost populations and their migration routes. Moreover, this study was performed on a rather small portion of the sugar maple range. Range‐wide sampling might provide different insights.

## CONFLICT OF INTEREST

None declared.

## AUTHOR CONTRIBUTIONS

N.G., F.T., and Y.B. conceived the ideas. N.G. collected samples, performed the laboratory work, analyzed the data, and led the writing of this manuscript. F.T. performed some statistical analysis and revised the manuscript.

## DATA ACCESSIBILITY

GenBank access numbers of microsatellites markers are listed in Table S1. Microsatellite data are in DRYAD entry doi: 10.5061/dryad.36634.
