# Supplementary material for: Influence of northern limit range on genetic diversity and structure in a widespread North American tree, sugar maple (Acer saccharum Marshall)
Source: Ecol Evol. 2018 Feb 8;8(5):2766–80. doi: 10.1002/ece3.3906 (PMC5838051; doi:10.1002/ece3.3906)
Supplement: Supplementary file 1 [file ECE3-8-2766-s001.doc]

*Ecology and Evolution*

**Supporting Information**

**Influence of northern limit range on genetic diversity and structure in a widespread North American tree, sugar maple (*Acer saccharum* Marshall)**

Noémie Graignic, Francine Tremblay and Yves Bergeron

**Table S1** Genetic variability estimates of microsatellite markers used in the study of sugar maple (*Acer saccharum* Marshall) in Québec.

| Locus | GenBank  access no. | M | Size range  (bp) | *A*_T_ | *A* | *H*_O_ | *H*_E_ | *F*_IS_ | References |
| --- | --- | --- | --- | --- | --- | --- | --- | --- | --- |
| SM11 | KC731552 | 3 | 178–200 | 13 | 6.3 | 0.575 | 0.629 | 0.083 | (Graignic *et al*. 2013) |
| SM14 | KC751436 | 4 | 70–120 | 22 | 14.3 | 0.773 | 0.888 | 0.130 | (Graignic *et al*. 2013) |
| SM21A | KC751437 | 4 | 173–243 | 31 | 14.8 | 0.780 | 0.866 | 0.096 | (Graignic *et al*. 2013) |
| SM22 | KC751438 | 2 | 293–325 | 19 | 12.6 | 0.658 | 0.890 | 0.248 | (Graignic *et al*. 2013) |
| SM27 | KC751440 | 4 | 242–260 | 9 | 6.4 | 0.548 | 0.722 | 0.227 | (Graignic *et al*. 2013) |
| SM29 | KC751441 | 4 | 272–307 | 12 | 6.4 | 0.598 | 0.724 | 0.164 | (Graignic *et al*. 2013) |
| SM34 | KC751442 | 3 | 118–171 | 26 | 13.7 | 0.819 | 0.854 | 0.041 | (Graignic *et al*. 2013) |
| SM36 | KC751443 | 5 | 146–196 | 21 | 11.3 | 0.795 | 0.844 | 0.057 | (Graignic *et al*. 2013) |
| SM37 | KC751444 | 2 | 174–200 | 14 | 7.9 | 0.633 | 0.669 | 0.061 | (Graignic *et al*. 2013) |
| SM42 | KC751445 | 1 | 90–135 | 20 | 7.9 | 0.726 | 0.789 | 0.080 | (Graignic *et al*. 2013) |
| SM47 | KC751446 | 2 | 201–225 | 11 | 6.2 | 0.428 | 0.634 | 0.328 | (Graignic *et al*. 2013) |
| SM51 | KC751447 | 1 | 269–290 | 9 | 4.5 | 0.395 | 0.482 | 0.172 | (Graignic *et al*. 2013) |
| SM53 | KC751448 | 5 | 287–310 | 10 | 3.1 | 0.435 | 0.520 | 0.156 | (Graignic *et al*. 2013) |
| SM55 | KC751449 | 2 | 246–276 | 14 | 9.1 | 0.567 | 0.678 | 0.166 | (Graignic *et al*. 2013) |
| SM56 | KC751450 | 3 | 287–299 | 6 | 4.7 | 0.433 | 0.618 | 0.278 | (Graignic *et al*. 2013) |
| SM60 | KC751452 | 3 | 231–243 | 4 | 2.8 | 0.393 | 0.457 | 0.137 | (Graignic *et al*. 2013) |
| Aop943 | EF531298 | 1 | 143–158 | 6 | 4.6 | 0.529 | 0.527 | -0.005 | (Segarra-Moragues *et al*. 2008)  (Graignic *et al*. 2013) |
| Am116 | AB303350 | 1 | 230–273 | 21 | 10.3 | 0.659 | 0.682 | 0.031 | (Kikuchi & Shibata 2008)  (Graignic *et al*. 2013) |

M, multiplexing arrangement; *A*_T_, total number of alleles; *A*, mean number of alleles; *H*_O_, mean observed heterozygosity; *H*_E_, mean expected heterozygosity; *F*_IS_, inbreeding coefficient.

Graignic N, Tremblay F, Bergeron Y (2013) Development of polymorphic nuclear microsatellite markers in sugar maple (*Acer saccharum* Marsh.) using cross-species transfer and SSR-enriched shotgun pyrosequencing. *Conservation Genetics Resources*, **5**, 845–848.

Kikuchi S, Shibata M (2008) Development of polymorphic microsatellite markers in *Acer mono* Maxim. *Molecular Ecology Notes*, **8**, 339–341.

Segarra-Moragues J, Gleiser G, González-Candelas F (2008) Isolation and characterization of microsatellite loci in *Acer opalus* (*Aceraceae*), a sexually-polymorphic tree, through an enriched genomic library. *Conservation Genetics*, **9**, 1059–1062.

**Table S2** Summary of *P*-values for the Hardy–Weinberg equilibrium using genepop (Markov chain parameters: 10 000 dememorizations, followed by 500 batches of 5000 iterations per batch).

|  | 1DA | 1DB | 1DC | 1DD | 1CA | 1CB | 1CC | 1CD | 2DA | 2DC | 2DD | 2CA | 2CB | 2CC | 2CD | 3DA | 3DB | 3DC | 3DD | 3CA | 3CB | 3CC | 3CD |
| --- | --- | --- | --- | --- | --- | --- | --- | --- | --- | --- | --- | --- | --- | --- | --- | --- | --- | --- | --- | --- | --- | --- | --- |
| SM11 | 0.0084 | 0.4624 | 0.5799 | 0.0734 | 0.0534 | 0.0360 | 0.3199 | 0.0337 | 0.2573 | 0.0423 | 0.0453 | 0.0395 | 0.0793 | 0.2054 | 0.5682 | 0.0083 | 0.9193 | 0.3279 | 0.5603 | 0.0012 | 0.3101 | 0.2052 | 0.5629 |
| SM14 | 0.1373 | 0.2148 | 0.0197 | 0.0768 | 0.0162 | 0.1430 | 0.0700 | 0.0014 | 0.2848 | 0.0018 | 0.6783 | 0.2159 | 0.2097 | 0.1402 | 0.3979 | 0.0040 | 0.0028 | 0.2548 | 0.5538 | 0.2092 | 0.2191 | 0.2174 | 0.3110 |
| SM21A | 0.0501 | 0.0786 | 0.3479 | 0.4064 | 0.0142 | 0.4691 | 0.7796 | 0.0002 | 0.0143 | 0.0373 | 0.3263 | 0.0331 | 0.7113 | 0.7279 | 0.0666 | 0.0342 | 0.5222 | 0.9107 | 0.5043 | **0.0000** | 0.1551 | 0.0316 | 0.0098 |
| SM22* | **0.0000** | 0.0173 | 0.2494 | 0.0024 | 0.0591 | 0.0362 | **0.0001** | **0.0000** | 0.0240 | 0.0010 | **0.0000** | 0.0003 | **0.0000** | 0.0002 | **0.0000** | **0.0000** | 0.2187 | 0.1322 | 0.5501 | 0.0062 | 0.0426 | **0.0000** | 0.5493 |
| SM27* | 0.0041 | 0.0061 | 0.1534 | 0.0186 | 0.0552 | 0.7882 | 0.7882 | **0.0000** | **0.0001** | **0.0000** | 0.0007 | 0.0193 | **0.0001** | 0.2009 | 0.0003 | **0.0000** | 0.1258 | 0.9890 | 0.7288 | 0.6601 | 0.6601 | **0.0000** | 0.7657 |
| SM29 | 0.3152 | 0.1248 | 0.5151 | 0.1278 | 0.4147 | 0.8772 | 0.2453 | 0.0099 | 0.0099 | 0.0011 | **0.0000** | 0.1115 | 0.0002 | 0.0209 | 0.0078 | **0.0001** | 0.2102 | 0.5353 | 0.0892 | 0.1776 | 0.3961 | **0.0000** | 0.5572 |
| SM34 | 0.3955 | 0.4077 | 0.5409 | 0.5499 | 0.1266 | 0.6818 | 0.6818 | 0.4333 | 0.0772 | 0.4617 | 0.0957 | 0.7941 | 0.1372 | 0.1517 | 0.3600 | 0.5645 | 0.4699 | 0.0625 | 0.2237 | 0.0851 | 0.0851 | 0.4614 | 0.2222 |
| SM36 | 0.6068 | 0.0593 | 0.2524 | 0.3244 | 0.1953 | 0.2661 | 0.7693 | 0.0374 | 0.8950 | 0.8950 | 0.4894 | 0.8393 | 0.7280 | 0.2362 | 0.6907 | 0.0096 | 0.1099 | 0.1302 | 0.7423 | 0.1329 | 0.0420 | 0.0420 | 0.9558 |
| SM37 | 0.3314 | 0.1111 | **0.0000** | 0.9321 | 0.8434 | 0.5688 | 0.1369 | 0.9524 | 0.0240 | 0.3770 | 0.1183 | 0.7735 | 0.2469 | 0.5934 | 0.0214 | 0.0024 | 0.5528 | 0.4821 | 0.5336 | 0.4371 | 0.4371 | 0.1365 | 0.9537 |
| SM42 | 0.0116 | 0.1733 | 0.1762 | 0.0175 | 0.5883 | 0.3816 | 0.3063 | 0.5406 | 0.3180 | 0.1948 | 0.6008 | 0.2435 | 0.9011 | 0.1066 | 0.0187 | 0.6354 | 0.3495 | 0.2544 | 0.0680 | 0.0680 | 0.3038 | 0.1323 | 0.1042 |
| SM47* | **0.0001** | **0.0001** | 0.0034 | **0.0000** | **0.0000** | 0.0148 | 0.0109 | 0.0109 | 0.0436 | 0.0344 | 0.0049 | 0.0005 | **0.0000** | 0.0005 | 0.0139 | **0.0000** | 0.8770 | 0.0748 | 0.0723 | **0.0001** | 0.0002 | **0.0000** | 0.0129 |
| SM51 | 0.4826 | 0.0358 | 1.0000 | 0.4594 | 0.2546 | 0.7119 | 0.9345 | 0.0168 | 0.8109 | 0.0084 | 0.1212 | 0.0879 | **0.0000** | 0.4727 | 0.0964 | **0.0000** | 1.0000 | 0.1210 | 0.9500 | 0.0009 | 1.0000 | 0.0013 | 0.6977 |
| SM53 | 0.0404 | 0.1050 | 0.4928 | 0.0198 | 0.2152 | 0.5037 | 0.0127 | 0.3701 | 0.5096 | 0.0001 | 0.0563 | 0.5266 | 0.0079 | 0.0002 | 0.1633 | 0.0793 | 0.4904 | 0.7101 | 0.5299 | 0.0407 | 0.1953 | 0.0050 | 0.3397 |
| SM55 | 0.3359 | 0.3359 | 1.0000 | 0.0649 | 0.7161 | 0.2880 | 0.4801 | 0.7785 | 0.4721 | **0.0001** | 0.0024 | 0.1680 | **0.0000** | 0.0046 | 0.0013 | 0.1252 | 0.5268 | 0.0038 | 0.2160 | 0.1743 | 0.0520 | 0.0017 | 0.1076 |
| SM56* | 0.0048 | 0.1476 | 0.2541 | 0.2859 | 0.1478 | 0.0094 | 0.9618 | 0.0232 | 0.1542 | **0.0000** | 0.0091 | 0.0056 | **0.0000** | 0.0014 | **0.0001** | **0.0000** | 0.2096 | 0.4728 | 0.4354 | **0.0001** | 0.2286 | **0.0000** | 0.5389 |
| SM60 | 0.5242 | 0.2293 | 0.6131 | 0.6162 | 0.4016 | 0.3282 | 1.0000 | 0.0186 | 0.0058 | 0.0018 | 0.0591 | 0.6880 | 0.0431 | 0.0199 | 0.6393 | 0.0005 | 0.8042 | 0.0092 | 0.1839 | 0.2218 | 0.1332 | 0.0012 | 0.4719 |
| Aop943 | 0.1550 | 0.1506 | 0.2770 | 0.2268 | 0.1954 | 0.9360 | 0.2570 | 0.4430 | 0.7489 | 0.4999 | 0.0912 | 0.1071 | 0.7181 | 0.7937 | 0.3839 | 0.8410 | 1.0000 | 1.0000 | 0.3578 | 0.0059 | 0.2053 | 0.5332 | 0.9946 |
| Am116 | 0.4107 | 0.1192 | 0.8810 | 0.1784 | 0.1821 | 0.2278 | 0.9833 | 0.1946 | 0.8047 | 0.1805 | 0.0236 | 0.4223 | 0.1911 | 0.6536 | 0.8772 | 0.6798 | 0.0297 | 0.4957 | 0.2049 | 0.1392 | 0.7788 | 0.4427 | 0.5740 |

Significant *P*-values after Bonferroni correction are in bold type. *indicates locus at which most populations show signs of deviation from HWE

**Table S3** Summary of null allele frequencies for each pair of loci and populations using freena.

|  | 1DA | 1DB | 1DC | 1DD | 1CA | 1CB | 1CC | 1CD | 2DA | 2DC | 2DD | 2CA | 2CB | 2CC | 2CD | 3DA | 3DB | 3DC | 3DD | 3CA | 3CB | 3CC | 3CD |
| --- | --- | --- | --- | --- | --- | --- | --- | --- | --- | --- | --- | --- | --- | --- | --- | --- | --- | --- | --- | --- | --- | --- | --- |
| SM11 | **0.102** | 0.000 | 0.013 | 0.000 | 0.062 | 0.020 | 0.000 | 0.079 | 0.021 | **0.101** | 0.058 | 0.054 | 0.066 | 0.031 | 0.038 | 0.052 | 0.000 | 0.038 | 0.000 | **0.101** | 0.036 | 0.000 | 0.003 |
| SM14 | 0.037 | 0.017 | 0.069 | 0.034 | 0.064 | 0.000 | 0.051 | 0.092 | 0.025 | 0.099 | 0.024 | 0.031 | 0.045 | 0.043 | 0.030 | **0.123** | **0.112** | 0.045 | 0.036 | 0.080 | 0.063 | 0.069 | 0.059 |
| SM21A | 0.093 | 0.051 | 0.029 | 0.022 | 0.071 | 0.000 | 0.013 | 0.098 | 0.000 | 0.097 | 0.067 | 0.063 | 0.000 | 0.000 | 0.094 | **0.107** | 0.000 | 0.000 | 0.000 | **0.144** | 0.000 | 0.048 | 0.009 |
| SM22* | **0.165** | 0.080 | 0.000 | **0.131** | 0.039 | 0.014 | **0.149** | **0.205** | **0.105** | **0.138** | **0.286** | 0.094 | **0.242** | **0.146** | **0.161** | **0.212** | 0.006 | 0.063 | 0.005 | 0.07 | 0.075 | **0.254** | 0.015 |
| SM27* | **0.156** | **0.147** | 0.026 | **0.110** | 0.046 | 0.041 | 0.023 | **0.213** | **0.172** | **0.230** | **0.156** | **0.111** | **0.204** | 0.084 | **0.142** | **0.192** | 0.000 | 0.000 | 0.000 | 0.094 | 0.031 | **0.232** | 0.000 |
| SM29 | 0.029 | 0.039 | 0.000 | 0.057 | 0.017 | 0.000 | 0.035 | 0.032 | **0.127** | **0.177** | **0.212** | 0.059 | **0.178** | 0.049 | **0.139** | **0.202** | 0.000 | 0.000 | 0.066 | 0.099 | 0.041 | **0.149** | 0.000 |
| SM34 | 0.003 | 0.000 | 0.000 | 0.000 | 0.039 | 0.000 | 0.000 | 0.065 | 0.000 | 0.000 | 0.034 | 0.000 | 0.018 | 0.000 | 0.000 | 0.026 | 0.017 | 0.031 | 0.015 | 0.085 | 0.044 | 0.000 | 0.000 |
| SM36 | 0.074 | 0.034 | 0.005 | 0.064 | 0.056 | 0.000 | 0.000 | 0.094 | 0.000 | 0.000 | 0.000 | 0.000 | 0.022 | 0.027 | 0.000 | 0.056 | 0.008 | 0.000 | 0.000 | 0.064 | 0.058 | 0.065 | 0.000 |
| SM37 | 0.077 | 0.078 | 0.000 | 0.000 | 0.009 | 0.000 | 0.000 | 0.000 | 0.022 | 0.000 | 0.076 | 0.000 | 0.046 | 0.000 | 0.071 | **0.127** | 0.005 | 0.034 | 0.000 | 0.000 | 0.036 | 0.096 | 0.000 |
| SM42 | **0.109** | 0.011 | 0.037 | 0.056 | 0.009 | 0.030 | 0.000 | 0.061 | 0.021 | 0.018 | 0.000 | 0.000 | 0.000 | 0.070 | 0.048 | 0.034 | 0.000 | 0.002 | 0.088 | 0.000 | 0.025 | 0.009 | 0.019 |
| SM47* | **0.157** | **0.176** | 0.000 | **0.218** | **0.178** | 0.085 | **0.156** | 0.093 | 0.042 | **0.113** | **0.160** | **0.143** | **0.179** | 0.095 | **0.137** | **0.173** | 0.009 | 0.096 | 0.085 | **0.161** | **0.162** | **0.231** | 0.090 |
| SM51 | 0.034 | 0.081 | 0.000 | 0.000 | 0.035 | 0.000 | 0.000 | **0.104** | 0.014 | **0.131** | **0.107** | 0.064 | **0.237** | 0.059 | **0.105** | **0.206** | 0.000 | 0.000 | 0.000 | **0.165** | 0.000 | **0.160** | 0.000 |
| SM53 | 0.084 | 0.058 | 0.041 | 0.000 | 0.000 | 0.000 | 0.021 | 0.046 | 0.038 | **0.161** | **0.112** | 0.035 | **0.172** | **0.187** | 0.076 | **0.105** | 0.040 | 0.000 | 0.000 | **0.113** | 0.079 | **0.167** | 0.000 |
| SM55 | 0.019 | 0.054 | 0.000 | 0.070 | 0.000 | 0.010 | 0.010 | 0.031 | 0.036 | **0.146** | **0.155** | 0.063 | **0.214** | 0.074 | 0.054 | 0.084 | 0.000 | **0.116** | 0.069 | 0.012 | 0.076 | **0.129** | 0.057 |
| SM56* | **0.125** | 0.083 | 0.000 | 0.080 | 0.082 | **0.116** | 0.010 | **0.135** | 0.081 | **0.212** | **0.152** | **0.150** | **0.226** | **0.155** | **0.145** | **0.313** | 0.000 | 0.000 | 0.000 | **0.191** | 0.076 | **0.257** | 0.000 |
| SM60 | 0.037 | 0.075 | 0.000 | 0.012 | 0.047 | 0.000 | 0.000 | **0.125** | **0.132** | **0.130** | **0.106** | 0.000 | **0.112** | **0.127** | 0.033 | **0.179** | 0.000 | 0.000 | 0.076 | 0.030 | 0.068 | **0.158** | 0.000 |
| Aop943 | 0.020 | 0.000 | 0.000 | 0.000 | 0.049 | 0.000 | 0.000 | 0.064 | 0.000 | 0.058 | 0.000 | 0.099 | 0.002 | 0.000 | 0.000 | 0.000 | 0.000 | 0.000 | 0.011 | 0.000 | 0.000 | 0.000 | 0.000 |
| Am116 | 0.051 | 0.080 | 0.000 | 0.046 | 0.025 | 0.000 | 0.000 | 0.000 | 0.000 | 0.034 | 0.000 | 0.000 | 0.033 | 0.000 | 0.000 | 0.000 | 0.035 | 0.000 | 0.000 | 0.048 | 0.000 | 0.002 | 0.000 |

High (≥ 10 %) frequency of null alleles are in bold type. *indicates locus at which most populations show signs of null allele

**Table S4** Genetic variability estimates of sugar maple (*Acer saccharum*) populations in Québec for mature trees, saplings or all individuals.

| Population ID | Population name | Cohorts | N | *A* | *A*_R_ | *A*_R_^*^ | *H*_O_ | *H*_E_ | *F*_IS_ | *F*_ST_ |
| --- | --- | --- | --- | --- | --- | --- | --- | --- | --- | --- |
| 1-D-A | Lac Labelle | IP | 40 | 7.8 | 6.6 | — | 0.532 | 0.684 | 0.222 | 0.020 |
|  |  | M | 20 | 6.2 | 5.1 | 4.9 | 0.566 | 0.668 | 0.152 | 0.019 |
|  |  | Sa | 20 | 6.8 | 5.3 | 5.3 | 0.500 | 0.702 | 0.287 | 0.020 |
| 1-D-B | Lac Okiwakamik | IP | 40 | 8.1 | 7.0 | — | 0.590 | 0.693 | 0.149 | 0.015 |
|  |  | M | 20 | 6.9 | 5.6 | 5.4 | 0.595 | 0.699 | 0.150 | 0.013 |
|  |  | Sa | 20 | 6.9 | 5.4 | 5.4 | 0.587 | 0.690 | 0.150 | 0.016 |
| 1-D-C | Rémigny | IP | 32 | 6.6 | 5.8 | — | 0.670 | 0.637 | -0.051 | 0.041 |
|  |  | M | 12 | 4.6 | 4.4 | 4.3 | 0.668 | 0.612 | -0.090 | 0.051 |
|  |  | Sa | 20 | 5.7 | 4.5 | 4.5 | 0.671 | 0.644 | -0.042 | 0.039 |
| 1-D-D | Lac de la Tour | IP | 40 | 7.9 | 6.9 | — | 0.608 | 0.700 | 0.131 | 0.017 |
|  |  | M | 20 | 6.8 | 5.4 | 5.2 | 0.591 | 0.700 | 0.156 | 0.020 |
|  |  | Sa | 20 | 6.6 | 5.2 | 5.2 | 0.625 | 0.693 | 0.099 | 0.020 |
| 1-C-A | Lac St Amand | IP | 40 | 8.7 | 7.5 | — | 0.620 | 0.704 | 0.120 | 0.011 |
|  |  | M | 20 | 6.9 | 5.8 | 5.6 | 0.638 | 0.702 | 0.092 | 0.011 |
|  |  | Sa | 20 | 7.3 | 5.5 | 5.5 | 0.604 | 0.709 | 0.147 | 0.009 |
| 1-C-B | Kipawa | IP | 39 | 8.0 | 7.2 | — | 0.697 | 0.702 | 0.007 | 0.013 |
|  |  | M | 19 | 6.8 | 5.8 | 5.6 | 0.705 | 0.689 | -0.023 | 0.016 |
|  |  | Sa | 20 | 7.1 | 5.5 | 5.5 | 0.687 | 0.707 | 0.029 | 0.012 |
| 1-C-C | Lac Six Milles | IP | 40 | 8.6 | 7.4 | — | 0.664 | 0.698 | 0.049 | 0.018 |
|  |  | M | 20 | 7.2 | 5.7 | 5.5 | 0.648 | 0.679 | 0.045 | 0.022 |
|  |  | Sa | 20 | 6.9 | 5.5 | 5.5 | 0.679 | 0.714 | 0.050 | 0.017 |
| 1-C-D | Lac Percival | IP | 40 | 8.5 | 7.3 | — | 0.546 | 0.696 | 0.216 | 0.011 |
|  |  | M | 20 | 6.6 | 5.8 | 5.5 | 0.547 | 0.715 | 0.207 | 0.011 |
|  |  | Sa | 20 | 7.2 | 5.4 | 5.4 | 0.535 | 0.686 | 0.220 | 0.009 |
| 2-D-A | Lac Pénobscot | IP | 40 | 8.1 | 7.1 | — | 0.623 | 0.709 | 0.121 | 0.019 |
|  |  | M | 20 | 6.8 | 5.5 | 5.3 | 0.617 | 0.700 | 0.119 | 0.025 |
|  |  | Sa | 20 | 6.8 | 5.5 | 5.5 | 0.630 | 0.713 | 0.117 | 0.018 |
| 2-D-C | Réservoir Mitchinamécus | IP | 40 | 8.5 | 7.4 | — | 0.548 | 0.715 | 0.234 | 0.014 |
|  |  | M | 20 | 7.6 | 6.2 | 5.9 | 0.577 | 0.726 | 0.205 | 0.005 |
|  |  | Sa | 20 | 6.4 | 5.3 | 5.3 | 0.520 | 0.706 | 0.264 | 0.022 |
| 2-D-D | Lac des Polonais | IP | 40 | 8.6 | 7.5 | — | 0.516 | 0.684 | 0.246 | 0.011 |
|  |  | M | 20 | 7.0 | 5.8 | 5.6 | 0.493 | 0.683 | 0.279 | 0.008 |
|  |  | Sa | 20 | 7.1 | 5.5 | 5.5 | 0.539 | 0.693 | 0.223 | 0.008 |
| 2-C-A | Montagne du Diable | IP | 40 | 8.2 | 7.1 | — | 0.596 | 0.684 | 0.129 | 0.012 |
|  |  | M | 20 | 6.9 | 5.5 | 5.3 | 0.614 | 0.655 | 0.062 | 0.017 |
|  |  | Sa | 20 | 6.8 | 5.3 | 5.3 | 0.579 | 0.705 | 0.180 | 0.010 |
| 2-C-B | Lac Ecuyer | IP | 40 | 8.6 | 7.3 | — | 0.505 | 0.698 | 0.276 | 0.012 |
|  |  | M | 20 | 6.9 | 5.7 | 5.4 | 0.495 | 0.692 | 0.284 | 0.009 |
|  |  | Sa | 20 | 7.0 | 5.4 | 5.4 | 0.519 | 0.707 | 0.266 | 0.013 |
| 2-C-C | Lac Marie-Lefranc | IP | 42 | 8.6 | 7.2 | — | 0.589 | 0.691 | 0.147 | 0.010 |
|  |  | M | 22 | 7.6 | 5.8 | 5.5 | 0.629 | 0.695 | 0.095 | 0.009 |
|  |  | Sa | 20 | 6.7 | 5.2 | 5.2 | 0.544 | 0.688 | 0.209 | 0.012 |
| 2-C-D | Lac de l'Ecluse | IP | 40 | 8.6 | 7.2 | — | 0.569 | 0.687 | 0.172 | 0.009 |
|  |  | M | 20 | 6.9 | 5.6 | 5.3 | 0.617 | 0.696 | 0.114 | 0.009 |
|  |  | Sa | 20 | 6.9 | 5.4 | 5.4 | 0.520 | 0.676 | 0.230 | 0.011 |
| 3-D-A | Lac Patrick | IP | 40 | 8.0 | 7.0 | — | 0.496 | 0.710 | 0.302 | 0.021 |
|  |  | M | 20 | 7.0 | 5.8 | 5.6 | 0.478 | 0.718 | 0.335 | 0.022 |
|  |  | Sa | 20 | 6.2 | 5.0 | 5.0 | 0.514 | 0.706 | 0.272 | 0.018 |
| 3-D-B | Fjord du Saguenay | IP | 40 | 7.8 | 7.0 | — | 0.681 | 0.687 | 0.008 | 0.017 |
|  |  | M | 20 | 6.9 | 5.7 | 5.5 | 0.696 | 0.687 | -0.012 | 0.015 |
|  |  | Sa | 20 | 6.9 | 5.5 | 5.5 | 0.667 | 0.690 | 0.034 | 0.018 |
| 3-D-C | Baie Eternité | IP | 40 | 7.4 | 6.3 | — | 0.639 | 0.661 | 0.033 | 0.026 |
|  |  | M | 20 | 6.4 | 5.2 | 5.0 | 0.630 | 0.659 | 0.045 | 0.020 |
|  |  | Sa | 20 | 6.2 | 5.0 | 5.0 | 0.649 | 0.667 | 0.027 | 0.027 |
| 3-D-D | Lac Edouard | IP | 40 | 8.3 | 6.5 | — | 0.656 | 0.694 | 0.054 | 0.019 |
|  |  | M | 20 | 6.5 | 5.5 | 5.3 | 0.681 | 0.705 | 0.035 | 0.017 |
|  |  | Sa | 20 | 6.2 | 5.1 | 5.1 | 0.613 | 0.681 | 0.074 | 0.021 |
| 3-C-A | Lac Paul | IP | 40 | 8.4 | 7.1 | — | 0.548 | 0.699 | 0.215 | 0.019 |
|  |  | M | 20 | 6.9 | 5.7 | 5.4 | 0.574 | 0.701 | 0.181 | 0.016 |
|  |  | Sa | 20 | 6.6 | 5.2 | 5.2 | 0.524 | 0.701 | 0.252 | 0.017 |
| 3-C-B | Lac Dickey | IP | 40 | 9.0 | 7.6 | — | 0.618 | 0.700 | 0.117 | 0.011 |
|  |  | M | 20 | 7.6 | 6.0 | 5.8 | 0.651 | 0.715 | 0.090 | 0.011 |
|  |  | Sa | 20 | 6.9 | 5.3 | 5.3 | 0.585 | 0.681 | 0.140 | 0.014 |
| 3-C-C | Lac Grandbois | IP | 40 | 8.0 | 6.9 | — | 0.499 | 0.691 | 0.278 | 0.012 |
|  |  | M | 20 | 6.8 | 5.6 | 5.4 | 0.479 | 0.691 | 0.307 | 0.008 |
|  |  | Sa | 20 | 6.4 | 5.1 | 5.1 | 0.515 | 0.694 | 0.258 | 0.014 |
| 3-C-D | Lac Larose | IP | 40 | 8.2 | 7.0 | — | 0.716 | 0.715 | -0.000 | 0.012 |
|  |  | M | 20 | 6.9 | 5.6 | 5.4 | 0.740 | 0.720 | -0.027 | 0.011 |
|  |  | Sa | 20 | 6.9 | 5.4 | 5.4 | 0.691 | 0.713 | 0.031 | 0.011 |
| Means |  |  | 40 | 8.2 | 7.0 | 5.3 | 0.597 | 0.693 | 0.138 | 0.016 |

IP, all individuals pooled; M, mature trees; Sa, saplings; N, sample size; *A*, mean number of alleles; *A*_R_, mean allelic richness; *H*_O_, mean observed heterozygosity; *H*_E_, mean expected heterozygosity; *F*_IS_, inbreeding coefficient; *F*_ST_, mean pairwise *F*_ST_. Means was determined using IP except for *A*_R_^*^.

**Table S5** Pairwise population *F*_ST_ of (a) all individuals pooled, (b) mature trees and (c) saplings of sugar maple (*Acer saccharum*) for all 23 sites in Québec below the diagonal. *P*-values are found above the diagonal. Significant values after adjusted nominal level (α = 0.05) for multiple comparisons are given in bold type.

Table S5 a)

|  | 1DA | 1DB | 1DC | 1DD | 1CA | 1CB | 1CC | 1CD | 2DA | 2DC | 2DD | 2CA | 2CB | 2CC | 2CD | 3DA | 3DB | 3DC | 3DD | 3CA | 3CB | 3CC | 3CD |
| --- | --- | --- | --- | --- | --- | --- | --- | --- | --- | --- | --- | --- | --- | --- | --- | --- | --- | --- | --- | --- | --- | --- | --- |
| 1DA | — | **0.0002** | **0.0002** | **0.0002** | **0.0002** | **0.0002** | **0.0002** | **0.0002** | **0.0002** | **0.0002** | 0.0057 | **0.0002** | **0.0002** | **0.0002** | **0.0002** | **0.0002** | **0.0002** | **0.0002** | **0.0002** | **0.0002** | **0.0002** | **0.0002** | **0.0002** |
| 1DB | 0.0163 | — | **0.0002** | **0.0002** | **0.0002** | **0.0002** | **0.0002** | 0.0172 | **0.0002** | **0.0002** | 0.2727 | **0.0002** | **0.0002** | **0.0002** | 0.0103 | **0.0002** | **0.0002** | **0.0002** | **0.0002** | **0.0002** | **0.0002** | 0.0012 | **0.0002** |
| 1DC | 0.0457 | 0.0376 | — | **0.0002** | **0.0002** | **0.0002** | **0.0002** | **0.0002** | **0.0002** | **0.0002** | **0.0002** | **0.0002** | **0.0002** | **0.0002** | **0.0002** | **0.0002** | **0.0002** | **0.0002** | **0.0002** | **0.0002** | **0.0002** | **0.0002** | **0.0002** |
| 1DD | 0.0210 | 0.0149 | 0.0546 | — | 0.0012 | **0.0002** | **0.0002** | **0.0002** | **0.0002** | **0.0002** | 0.0008 | **0.0002** | 0.0006 | **0.0002** | 0.0004 | **0.0002** | **0.0002** | **0.0002** | **0.0002** | **0.0002** | **0.0002** | **0.0002** | **0.0002** |
| 1CA | 0.0122 | 0.0072 | 0.0356 | 0.0060 | — | **0.0002** | **0.0002** | 0.0858 | **0.0002** | 0.0004 | 0.1654 | **0.0002** | **0.0002** | **0.0002** | 0.0006 | **0.0002** | **0.0002** | **0.0002** | **0.0002** | **0.0002** | **0.0002** | 0.0295 | **0.0002** |
| 1CB | 0.0198 | 0.0112 | 0.0335 | 0.0136 | 0.0044 | — | 0.0010 | 0.0061 | **0.0002** | 0.0004 | 0.0016 | **0.0002** | **0.0002** | **0.0002** | 0.0055 | **0.0002** | **0.0002** | **0.0002** | **0.0002** | **0.0002** | **0.0002** | 0.0008 | **0.0002** |
| 1CC | 0.0209 | 0.0131 | 0.0408 | 0.0147 | 0.0047 | 0.0079 | — | 0.0544 | **0.0002** | **0.0002** | 0.0638 | **0.0002** | **0.0002** | **0.0002** | **0.0002** | **0.0002** | **0.0002** | **0.0002** | **0.0002** | **0.0002** | **0.0002** | **0.0002** | **0.0002** |
| 1CD | 0.0140 | 0.0033 | 0.0303 | 0.0130 | 0.0012 | 0.0000 | 0.0029 | — | **0.0002** | 0.0008 | 0.1132 | 0.0053 | 0.0101 | 0.0332 | 0.0103 | **0.0002** | **0.0002** | **0.0002** | **0.0002** | **0.0002** | 0.0004 | 0.0190 | **0.0002** |
| 2DA | 0.0271 | 0.0168 | 0.0505 | 0.0197 | 0.0139 | 0.019 | 0.0211 | 0.0167 | — | **0.0002** | 0.0022 | **0.0002** | **0.0002** | **0.0002** | **0.0002** | **0.0002** | **0.0002** | **0.0002** | **0.0002** | **0.0002** | **0.0002** | **0.0002** | **0.0002** |
| 2DC | 0.0144 | 0.0150 | 0.0344 | 0.0128 | 0.0127 | 0.0108 | 0.0175 | 0.0101 | 0.0214 | — | 0.0708 | 0.0008 | 0.0091 | **0.0002** | 0.0334 | **0.0002** | **0.0002** | **0.0002** | **0.0002** | **0.0002** | **0.0002** | 0.0008 | **0.0002** |
| 2DD | 0.0174 | 0.0079 | 0.0329 | 0.0112 | 0.0051 | 0.0093 | 0.0132 | 0.0073 | 0.0103 | 0.0089 | — | 0.1192 | 0.6575 | 0.1441 | 0.6166 | 0.0018 | 0.0028 | **0.0002** | 0.0006 | 0.0077 | 0.0935 | 0.3455 | 0.1191 |
| 2CA | 0.0141 | 0.0137 | 0.0362 | 0.0146 | 0.0096 | 0.0094 | 0.0147 | 0.0078 | 0.0236 | 0.0051 | 0.0038 | — | 0.0065 | 0.0595 | 0.0014 | **0.0002** | **0.0002** | **0.0002** | **0.0002** | **0.0002** | 0.0004 | 0.0063 | 0.0004 |
| 2CB | 0.0208 | 0.0113 | 0.0427 | 0.0082 | 0.0086 | 0.0136 | 0.0242 | 0.0120 | 0.0148 | 0.0039 | 0.0009 | 0.0075 | — | 0.0059 | 0.0293 | **0.0002** | **0.0002** | **0.0002** | **0.0002** | **0.0002** | 0.0129 | 0.0316 | 0.0004 |
| 2CC | 0.0179 | 0.0109 | 0.0455 | 0.0119 | 0.0078 | 0.0089 | 0.0168 | 0.0061 | 0.0161 | 0.0112 | 0.0051 | 0.0022 | 0.0054 | — | 0.1777 | **0.0002** | **0.0002** | **0.0002** | **0.0002** | **0.0002** | 0.0020 | 0.2374 | **0.0002** |
| 2CD | 0.0138 | 0.0098 | 0.0327 | 0.0103 | 0.0047 | 0.0021 | 0.0130 | 0.0050 | 0.0156 | 0.0045 | 0.0052 | 0.0077 | 0.0060 | -0.0003 | — | 0.0006 | **0.0002** | **0.0002** | **0.0002** | **0.0002** | 0.0067 | 0.1186 | 0.0006 |
| 3DA | 0.029 | 0.0202 | 0.0519 | 0.0316 | 0.0211 | 0.0207 | 0.0267 | 0.0150 | 0.0107 | 0.0244 | 0.0223 | 0.0307 | 0.018 | 0.0150 | 0.0097 | — | **0.0002** | **0.0002** | **0.0002** | **0.0002** | **0.0002** | 0.0004 | **0.0002** |
| 3DB | 0.0191 | 0.0251 | 0.0574 | 0.0181 | 0.0158 | 0.0188 | 0.0278 | 0.0186 | 0.0179 | 0.0170 | 0.0116 | 0.0115 | 0.0125 | 0.0075 | 0.0120 | 0.0205 | — | **0.0002** | **0.0002** | **0.0002** | **0.0002** | **0.0002** | **0.0002** |
| 3DC | 0.0380 | 0.0332 | 0.0613 | 0.0320 | 0.0223 | 0.0217 | 0.0379 | 0.0265 | 0.0334 | 0.0316 | 0.0258 | 0.0163 | 0.0198 | 0.0121 | 0.0230 | 0.0335 | 0.0185 | — | **0.0002** | **0.0002** | **0.0002** | **0.0002** | **0.0002** |
| 3DD | 0.0266 | 0.029 | 0.038 | 0.0310 | 0.0172 | 0.0143 | 0.0225 | 0.0184 | 0.0254 | 0.0131 | 0.0138 | 0.0144 | 0.0197 | 0.0133 | 0.0074 | 0.0187 | 0.0196 | 0.0238 | — | **0.0002** | **0.0002** | **0.0002** | **0.0002** |
| 3CA | 0.0298 | 0.0205 | 0.0578 | 0.0146 | 0.0153 | 0.0213 | 0.0197 | 0.0140 | 0.0227 | 0.0200 | 0.0093 | 0.0125 | 0.0072 | 0.0118 | 0.0189 | 0.0242 | 0.0112 | 0.0224 | 0.0297 | — | **0.0002** | **0.0002** | **0.0002** |
| 3CB | 0.0175 | 0.0133 | 0.0448 | 0.0112 | 0.0095 | 0.0125 | 0.0166 | 0.0110 | 0.0097 | 0.0089 | 0.0075 | 0.0076 | 0.0055 | 0.0034 | 0.0018 | 0.0150 | 0.0100 | 0.0180 | 0.0126 | 0.0130 | — | 0.2328 | **0.0002** |
| 3CC | 0.0193 | 0.0098 | 0.0415 | 0.0164 | 0.0041 | 0.0079 | 0.0167 | 0.0086 | 0.0157 | 0.0174 | 0.0037 | 0.0073 | 0.0086 | 0.0007 | 0.0033 | 0.0193 | 0.0154 | 0.0195 | 0.0170 | 0.0158 | 0.0042 | — | 0.0055 |
| 3CD | 0.0165 | 0.0163 | 0.0397 | 0.0180 | 0.0062 | 0.0085 | 0.0135 | 0.0084 | 0.0192 | 0.0108 | 0.0141 | 0.0079 | 0.0112 | 0.005 | 0.0045 | 0.0137 | 0.0118 | 0.0181 | 0.0096 | 0.0173 | 0.0068 | 0.0090 | — |

Table S5 b)

|  | 1DA | 1DB | 1DC | 1DD | 1CA | 1CB | 1CC | 1CD | 2DA | 2DC | 2DD | 2CA | 2CB | 2CC | 2CD | 3DA | 3DB | 3DC | 3DD | 3CA | 3CB | 3CC | 3DD |
| --- | --- | --- | --- | --- | --- | --- | --- | --- | --- | --- | --- | --- | --- | --- | --- | --- | --- | --- | --- | --- | --- | --- | --- |
| 1DA | — | 0.0055 | **0.0002** | 0.0004 | 0.0055 | 0.0006 | 0.0004 | 0.0229 | **0.0002** | 0.0820 | 0.1648 | 0.0132 | 0.0095 | 0.0014 | 0.0030 | 0.0012 | **0.0002** | **0.0002** | **0.0002** | **0.0002** | 0.0004 | 0.0190 | **0.0002** |
| 1DB | 0.0148 | — | **0.0002** | 0.0012 | 0.0004 | 0.0010 | **0.0002** | 0.4482 | 0.0012 | 0.0176 | 0.5071 | 0.1795 | 0.0172 | 0.0024 | 0.1457 | 0.0024 | **0.0002** | **0.0002** | **0.0002** | **0.0002** | 0.0034 | 0.1287 | 0.0069 |
| 1DC | 0.0453 | 0.0443 | — | **0.0002** | **0.0002** | 0.0004 | **0.0002** | **0.0002** | **0.0002** | 0.0006 | 0.0087 | **0.0002** | 0.0008 | **0.0002** | **0.0002** | 0.0004 | **0.0002** | **0.0002** | **0.0002** | **0.0002** | **0.0002** | 0.0004 | **0.0002** |
| 1DD | 0.0252 | 0.0192 | 0.0788 | — | 0.1411 | 0.0010 | 0.0006 | 0.0162 | **0.0002** | 0.0464 | 0.0354 | 0.0026 | 0.0032 | 0.0036 | 0.0063 | 0.0004 | 0.0004 | **0.0002** | **0.0002** | 0.0004 | 0.0004 | 0.1089 | 0.0006 |
| 1CA | 0.0151 | 0.0123 | 0.0519 | 0.0047 | — | 0.0018 | 0.0026 | 0.1478 | 0.0010 | 0.2743 | 0.1901 | 0.0014 | 0.1553 | 0.0259 | 0.0022 | 0.0004 | 0.0099 | **0.0002** | **0.0002** | 0.0004 | 0.0132 | 0.4461 | 0.0004 |
| 1CB | 0.0206 | 0.0184 | 0.0509 | 0.0162 | 0.0127 | — | 0.0113 | 0.0589 | **0.0002** | 0.2832 | 0.0893 | 0.0425 | 0.0107 | 0.0022 | 0.1755 | 0.0004 | 0.0032 | 0.0006 | **0.0002** | 0.0014 | 0.0010 | 0.1140 | 0.0055 |
| 1CC | 0.0219 | 0.0201 | 0.0601 | 0.0273 | 0.0031 | 0.0146 | — | 0.0870 | **0.0002** | 0.0028 | 0.1488 | 0.0105 | 0.0109 | **0.0002** | 0.0008 | 0.0010 | **0.0002** | **0.0002** | **0.0002** | **0.0002** | **0.0002** | 0.0004 | **0.0002** |
| 1CD | 0.0110 | -0.0025 | 0.0450 | 0.0141 | 0.0021 | 0.0072 | 0.0049 | — | 0.0279 | 0.3599 | 0.2694 | 0.1852 | 0.2423 | 0.1553 | 0.1018 | 0.2239 | 0.0026 | 0.0012 | 0.0004 | 0.0008 | 0.0014 | 0.0334 | 0.0036 |
| 2DA | 0.0350 | 0.0182 | 0.0707 | 0.0285 | 0.0223 | 0.0279 | 0.0317 | 0.0220 | — | 0.0020 | 0.0492 | 0.0010 | 0.0042 | 0.0006 | 0.0018 | 0.0008 | 0.0006 | **0.0002** | **0.0002** | **0.0002** | 0.0030 | 0.0063 | **0.0002** |
| 2DC | 0.0063 | 0.0056 | 0.0422 | 0.0041 | 0.0015 | 0.0029 | 0.0141 | -0.0015 | 0.0208 | — | 0.4636 | 0.5856 | 0.3895 | 0.1708 | 0.4549 | 0.0044 | 0.0447 | **0.0002** | 0.0255 | 0.0089 | 0.0245 | 0.0433 | 0.0808 |
| 2DD | 0.0063 | 0.0044 | 0.0393 | 0.0079 | 0.0010 | 0.0126 | 0.0142 | 0.0079 | 0.0232 | -0.0077 | — | 0.3318 | 0.7573 | 0.3767 | 0.2723 | 0.1020 | 0.2652 | 0.0259 | 0.0789 | 0.2658 | 0.1172 | 0.2757 | 0.3387 |
| 2CA | 0.0180 | 0.0059 | 0.0547 | 0.0200 | 0.0155 | 0.0148 | 0.0238 | 0.0136 | 0.0374 | -0.0010 | 0.0059 | — | 0.3557 | 0.1026 | 0.0911 | 0.0026 | 0.0212 | 0.0004 | 0.0004 | 0.0008 | 0.0202 | 0.0263 | 0.0129 |
| 2CB | 0.0115 | 0.0062 | 0.0535 | 0.0087 | 0.0047 | 0.0197 | 0.0268 | 0.0087 | 0.0200 | -0.0059 | -0.0067 | 0.0045 | — | 0.0759 | 0.0457 | 0.0111 | 0.2162 | 0.0178 | 0.0130 | 0.0206 | 0.1239 | 0.3186 | 0.0777 |
| 2CC | 0.0105 | 0.0136 | 0.0507 | 0.0122 | 0.0049 | 0.0098 | 0.0226 | 0.0059 | 0.0213 | -0.0035 | 0.0012 | 0.0076 | 0.0021 | — | 0.4644 | 0.0593 | 0.2966 | 0.0095 | 0.0012 | 0.0006 | 0.0016 | 0.6273 | 0.0030 |
| 2CD | 0.0142 | 0.0057 | 0.0578 | 0.0104 | 0.0070 | -0.0027 | 0.0180 | 0.0050 | 0.0131 | -0.0065 | 0.0078 | 0.0073 | 0.0050 | -0.0056 | — | 0.0459 | 0.0725 | 0.0002 | 0.0008 | 0.0020 | 0.0010 | 0.2974 | 0.0156 |
| 3DA | 0.0344 | 0.0187 | 0.071 | 0.0376 | 0.0233 | 0.0220 | 0.0298 | 0.0067 | 0.0032 | 0.0169 | 0.0283 | 0.0444 | 0.0185 | 0.0168 | 0.0089 | — | 0.0073 | **0.0002** | 0.0004 | 0.0006 | 0.0160 | 0.1285 | 0.0030 |
| 3DB | 0.0210 | 0.0229 | 0.0650 | 0.0124 | 0.0117 | 0.0232 | 0.0302 | 0.0154 | 0.0196 | 0.0059 | 0.0056 | 0.0160 | 0.0027 | -0.0009 | 0.0067 | 0.0202 | — | 0.0091 | 0.0008 | 0.0249 | 0.0073 | 0.0842 | 0.1026 |
| 3DC | 0.0352 | 0.0200 | 0.0615 | 0.0237 | 0.0169 | 0.0265 | 0.0347 | 0.0195 | 0.0350 | 0.0161 | 0.0194 | 0.0192 | 0.0054 | 0.0055 | 0.0149 | 0.0310 | 0.0089 | — | 0.0004 | **0.0002** | 0.0059 | 0.0757 | 0.0006 |
| 3DD | 0.0245 | 0.0197 | 0.0435 | 0.0305 | 0.0217 | 0.0115 | 0.0293 | 0.0155 | 0.0307 | 0.0019 | 0.0077 | 0.0239 | 0.0155 | 0.0064 | 0.0079 | 0.0185 | 0.0166 | 0.0189 | — | **0.0002** | 0.0158 | 0.0028 | 0.0047 |
| 3CA | 0.0250 | 0.0146 | 0.0636 | 0.0107 | 0.0090 | 0.0235 | 0.0217 | 0.0132 | 0.0270 | 0.0056 | -0.0014 | 0.0189 | 0.0003 | 0.0134 | 0.0157 | 0.0223 | 0.0077 | 0.0207 | 0.0184 | — | **0.0002** | 0.0148 | 0.0018 |
| 3CB | 0.0174 | 0.0065 | 0.0412 | 0.0217 | 0.0125 | 0.0129 | 0.0218 | 0.0133 | 0.0185 | 0.0011 | 0.0065 | 0.013 | 0.0028 | 0.0065 | 0.0042 | 0.0156 | 0.0141 | 0.0114 | 0.0059 | 0.0121 | — | 0.5146 | 0.0300 |
| 3CC | 0.0066 | 0.0089 | 0.0387 | 0.0159 | -0.0024 | 0.0041 | 0.0114 | 0.0095 | 0.0196 | 0.0040 | -0.0034 | 0.0118 | 0.0030 | -0.0044 | 0.0002 | 0.0148 | 0.0097 | 0.0099 | 0.0117 | 0.0115 | 0.0004 | — | 0.4038 |
| 3CD | 0.0196 | 0.0074 | 0.0440 | 0.0204 | 0.0085 | 0.0114 | 0.0147 | 0.0066 | 0.0208 | -0.0005 | 0.0138 | 0.0148 | 0.0071 | 0.0058 | 0.0018 | 0.0082 | 0.0123 | 0.0162 | 0.0056 | 0.014 | 0.0008 | 0.0005 | — |

Table S5 c)

|  | 1DA | 1DB | 1DC | 1DD | 1CA | 1CB | 1CC | 1CD | 2DA | 2DC | 2DD | 2CA | 2CB | 2CC | 2CD | 3DA | 3DB | 3DC | 3DD | 3CA | 3CB | 3CC | 3CD |
| --- | --- | --- | --- | --- | --- | --- | --- | --- | --- | --- | --- | --- | --- | --- | --- | --- | --- | --- | --- | --- | --- | --- | --- |
| 1DA | — | 0.0038 | **0.0002** | **0.0002** | 0.1348 | 0.0014 | **0.0002** | 0.0376 | **0.0002** | 0.0004 | 0.0629 | 0.0047 | 0.0144 | 0.0014 | 0.0032 | 0.0004 | 0.0004 | **0.0002** | **0.0002** | 0.0004 | 0.0004 | **0.0002** | **0.0002** |
| 1DB | 0.0188 | — | **0.0002** | 0.0480 | 0.1887 | 0.0360 | 0.2668 | 0.6065 | 0.0008 | 0.0018 | 0.4737 | **0.0002** | 0.1935 | 0.1170 | 0.2332 | 0.0004 | **0.0002** | **0.0002** | **0.0002** | 0.0061 | **0.0002** | 0.0215 | 0.0008 |
| 1DC | 0.0438 | 0.0297 | — | **0.0002** | **0.0002** | **0.0002** | **0.0002** | 0.0004 | **0.0002** | **0.0002** | 0.0006 | **0.0002** | **0.0002** | **0.0002** | **0.0002** | **0.0002** | **0.0002** | **0.0002** | **0.0002** | **0.0002** | **0.0002** | **0.0002** | **0.0002** |
| 1DD | 0.0238 | 0.0061 | 0.0529 | — | 0.0063 | 0.0004 | 0.0613 | 0.0190 | **0.0002** | 0.0024 | 0.0125 | 0.0004 | 0.0295 | 0.0004 | 0.0061 | 0.0010 | **0.0002** | **0.0002** | **0.0002** | 0.0034 | **0.0002** | 0.0004 | **0.0002** |
| 1CA | 0.0018 | 0.0049 | 0.0270 | 0.0109 | — | 0.0381 | 0.0077 | 0.8992 | 0.0004 | 0.0051 | 0.2298 | 0.0682 | 0.0340 | 0.0658 | 0.3595 | 0.0016 | 0.0006 | 0.0008 | **0.0002** | 0.0044 | 0.0217 | 0.1710 | 0.0075 |
| 1CB | 0.0149 | 0.0062 | 0.0345 | 0.0198 | 0.0031 | — | 0.1872 | 0.9055 | 0.0012 | 0.0014 | 0.1316 | 0.0253 | 0.0059 | 0.1233 | 0.1490 | 0.0010 | **0.0002** | **0.0002** | **0.0002** | 0.0210 | 0.0010 | 0.1283 | 0.0069 |
| 1CC | 0.0217 | 0.0119 | 0.0392 | 0.0095 | 0.0081 | 0.0073 | — | 0.4842 | 0.0012 | 0.0050 | 0.0743 | **0.0002** | 0.0040 | 0.0097 | 0.0354 | 0.0059 | **0.0002** | **0.0002** | **0.0002** | 0.0030 | 0.0012 | 0.0028 | 0.0014 |
| 1CD | 0.0162 | 0.0018 | 0.0242 | 0.0101 | -0.0030 | -0.0014 | 0.0010 | — | 0.0047 | 0.0166 | 0.8344 | 0.2150 | 0.3719 | 0.7710 | 0.5812 | 0.0370 | 0.0049 | 0.0030 | 0.0004 | 0.2372 | 0.2937 | 0.3504 | 0.0461 |
| 2DA | 0.0233 | 0.0132 | 0.0440 | 0.0245 | 0.0067 | 0.0132 | 0.0147 | 0.0133 | — | **0.0002** | 0.0407 | **0.0002** | 0.0457 | **0.0002** | 0.0012 | 0.0223 | **0.0002** | 0.0004 | **0.0002** | **0.0002** | **0.0002** | 0.0075 | **0.0002** |
| 2DC | 0.0176 | 0.0267 | 0.0345 | 0.0239 | 0.0154 | 0.0220 | 0.0169 | 0.0225 | 0.0255 | — | 0.0617 | 0.0006 | 0.0251 | 0.0223 | 0.0712 | 0.0008 | **0.0002** | **0.0002** | **0.0002** | 0.0004 | 0.0060 | 0.0065 | 0.0006 |
| 2DD | 0.0190 | 0.0038 | 0.0269 | 0.0097 | 0.0041 | 0.0058 | 0.0119 | -0.0030 | 0.0054 | 0.0219 | — | 0.0551 | 0.5314 | 0.2913 | 0.9476 | 0.1168 | 0.0026 | 0.0004 | 0.0573 | 0.0453 | 0.4700 | 0.1012 | 0.3822 |
| 2CA | 0.0092 | 0.0225 | 0.0330 | 0.0223 | 0.0033 | 0.0056 | 0.0164 | 0.0052 | 0.0159 | 0.0109 | 0.0019 | — | 0.0779 | 0.4449 | 0.0646 | **0.0002** | 0.0004 | **0.0002** | 0.0004 | 0.0636 | 0.0032 | 0.0217 | 0.0320 |
| 2CB | 0.0246 | 0.0105 | 0.0333 | 0.0121 | 0.0061 | 0.0126 | 0.0193 | 0.0086 | 0.0045 | 0.0107 | 0.0002 | 0.0076 | — | 0.4210 | 0.3441 | 0.0310 | 0.0036 | 0.0111 | **0.0002** | 0.0192 | 0.0431 | 0.0073 | 0.0057 |
| 2CC | 0.0236 | 0.0111 | 0.0471 | 0.0157 | 0.0117 | 0.0126 | 0.0151 | 0.0024 | 0.0184 | 0.0215 | 0.0013 | 0.0019 | 0.0069 | — | 0.3609 | 0.0085 | 0.0304 | 0.0154 | **0.0002** | 0.1636 | 0.2259 | 0.2127 | 0.1658 |
| 2CD | 0.0159 | 0.0122 | 0.0242 | 0.0198 | -0.0003 | 0.0098 | 0.0159 | 0.0010 | 0.0192 | 0.0145 | -0.0021 | 0.0073 | 0.0099 | 0.0132 | — | 0.0178 | 0.0014 | **0.0002** | 0.0449 | 0.0004 | 0.5028 | 0.3763 | 0.0385 |
| 3DA | 0.0223 | 0.0223 | 0.0391 | 0.0313 | 0.0159 | 0.0217 | 0.0185 | 0.0137 | 0.0141 | 0.0239 | 0.0066 | 0.0235 | 0.0086 | 0.008 | 0.0116 | — | 0.0018 | 0.0006 | **0.0002** | 0.0018 | 0.0004 | 0.0200 | 0.0030 |
| 3DB | 0.0153 | 0.0249 | 0.0566 | 0.0241 | 0.0151 | 0.0218 | 0.0330 | 0.0160 | 0.0188 | 0.0299 | 0.0107 | 0.0089 | 0.0151 | 0.0104 | 0.0169 | 0.0162 | — | **0.0002** | **0.0002** | 0.0285 | 0.0010 | **0.0002** | 0.0113 |
| 3DC | 0.0371 | 0.0447 | 0.0688 | 0.0413 | 0.0227 | 0.0175 | 0.0322 | 0.0267 | 0.0327 | 0.0413 | 0.0257 | 0.0117 | 0.0268 | 0.0136 | 0.0342 | 0.0284 | 0.0224 | — | **0.0002** | 0.0079 | **0.0002** | **0.0002** | **0.0002** |
| 3DD | 0.0253 | 0.0342 | 0.0391 | 0.0334 | 0.0121 | 0.0178 | 0.0243 | 0.0182 | 0.0277 | 0.0238 | 0.0093 | 0.0105 | 0.0266 | 0.0218 | 0.0040 | 0.0218 | 0.0225 | 0.0259 | — | **0.0002** | **0.0002** | 0.0006 | **0.0002** |
| 3CA | 0.027 | 0.0192 | 0.0590 | 0.0181 | 0.0161 | 0.0139 | 0.0189 | 0.0102 | 0.0200 | 0.0313 | 0.0081 | 0.0057 | 0.0106 | 0.0069 | 0.0220 | 0.0174 | 0.0083 | 0.0135 | 0.0376 | — | 0.0872 | 0.0312 | 0.0105 |
| 3CB | 0.0200 | 0.0185 | 0.0497 | 0.0145 | 0.0090 | 0.0161 | 0.0242 | 0.0092 | 0.0123 | 0.0230 | 0.0081 | 0.0112 | 0.0146 | 0.0050 | -0.0004 | 0.0155 | 0.0064 | 0.0248 | 0.0171 | 0.0092 | — | 0.5057 | 0.0014 |
| 3CC | 0.0249 | 0.0080 | 0.0450 | 0.0238 | 0.0058 | 0.0028 | 0.0252 | 0.0065 | 0.0181 | 0.0277 | 0.0054 | 0.0051 | 0.0129 | 0.0025 | 0.0052 | 0.0162 | 0.0165 | 0.0273 | 0.0251 | 0.0093 | 0.0017 | — | 0.0061 |
| 3CD | 0.0086 | 0.0199 | 0.0384 | 0.0221 | 0.0018 | 0.0044 | 0.0089 | 0.0086 | 0.0174 | 0.019 | 0.0067 | 0.0016 | 0.0138 | 0.0055 | 0.0038 | 0.0102 | 0.0114 | 0.0104 | 0.0095 | 0.0134 | 0.0111 | 0.0155 | — |

**Table S6** Comparison of mean genetic variability estimates between populations of zones, transects, and zones within each transect of sugar maple (*Acer saccharum*) in Québec for mature trees, saplings and all individuals.

| Cohorts | Genetic  Indices | Zones | | | Transects | | | | | | | | | | | | |
| --- | --- | --- | --- | --- | --- | --- | --- | --- | --- | --- | --- | --- | --- | --- | --- | --- | --- |
|  |  | D | C | *P*-value | 1 | 2 | 3 | *P*-value | 1 | | | 2 | | | 3 | | |
|  |  |  |  |  |  |  |  |  | Zone | | | Zone | | | Zone | | |
|  |  |  |  |  |  |  |  |  | D | C | *P*-value | D | C | *P*-value | D | C | *P*-value |
| IP | *A*_R_ | 6.840 | 7.246 | **0.0080** | 6.970 | 7.272 | 6.941 | 0.2450 | 6.601 | 7.339 | **0.0100** | 7.338 | 7.222 | 0.7380 | 6.707 | 7.176 | 0.0880 |
|  | *H*_O_ | 0.600 | 0.600 | 0.9950 | 0.616 | 0.567 | 0.611 | 0.2610 | 0.597 | 0.634 | 0.4460 | 0.566 | 0.567 | 0.9870 | 0.623 | 0.599 | 0.5800 |
|  | *H*_E_ | 0.689 | 0.698 | 0.2110 | 0.691 | 0.695 | 0.694 | 0.8610 | 0.680 | 0.701 | 0.0780 | 0.703 | 0.690 | 0.2630 | 0.687 | 0.701 | 0.1960 |
|  | *F*_IS_ | 0.129 | 0.140 | 0.7730 | 0.108 | 0.184 | 0.120 | 0.2520 | 0.122 | 0.095 | 0.6760 | 0.194 | 0.178 | 0.8380 | 0.093 | 0.146 | 0.4230 |
|  | *F*_ST_ | 0.025 | 0.009 | **0.0010** | 0.017 | 0.009 | 0.016 | 0.4630 | 0.030 | 0.004 | **0.0050** | 0.014 | 0.005 | 0.3910 | 0.022 | 0.011 | 0.2490 |
| M | *A*_R_ | 5.464 | 5.707 | 0.1000 | 5.458 | 5.708 | 5.621 | 0.4100 | 5.138 | 5.779 | **0.0060** | 5.816 | 5.626 | 0.4660 | 5.526 | 5.716 | 0.4430 |
|  | *H*_O_ | 0.603 | 0.616 | 0.6670 | 0.620 | 0.583 | 0.622 | 0.4900 | 0.599 | 0.641 | 0.4030 | 0.568 | 0.593 | 0.6630 | 0.627 | 0.616 | 0.8430 |
|  | *H*_E_ | 0.698 | 0.697 | 0.4020 | 0.687 | 0.693 | 0.699 | 0.4730 | 0.676 | 0.696 | 0.1990 | 0.703 | 0.686 | 0.3010 | 0.692 | 0.707 | 0.2940 |
|  | *F*_IS_ | 0.126 | 0.116 | 0.8260 | 0.097 | 0.158 | 0.111 | 0.5220 | 0.114 | 0.079 | 0.6400 | 0.193 | 0.135 | 0.4870 | 0.094 | 0.129 | 0.6410 |
|  | *F*_ST_ | 0.023 | 0.009 | **0.0250** | 0.021 | 0.007 | 0.013 | 0.2540 | 0.034 | 0.007 | **0.0280** | 0.013 | 0.003 | 0.4560 | 0.019 | 0.006 | 0.3290 |
| Sa | *A*_R_ | 5.200 | 5.343 | 0.1730 | 5.271 | 5.382 | 5.185 | 0.2730 | 5.078 | 5.464 | **0.0200** | 5.457 | 5.327 | 0.4790 | 5.130 | 5.240 | 0.5410 |
|  | *H*_O_ | 0.598 | 0.585 | 0.6370 | 0.612 | 0.551 | 0.601 | 0.1610 | 0.596 | 0.627 | 0.5320 | 0.565 | 0.541 | 0.6510 | 0.620 | 0.582 | 0.4260 |
|  | *H*_E_ | 0.689 | 0.698 | 0.1960 | 0.692 | 0.697 | 0.692 | 0.7790 | 0.682 | 0.703 | 0.0650 | 0.703 | 0.693 | 0.4270 | 0.685 | 0.698 | 0.2950 |
|  | *F*_IS_ | 0.132 | 0.163 | 0.4690 | 0.116 | 0.210 | 0.131 | 0.1440 | 0.126 | 0.109 | 0.8110 | 0.197 | 0.219 | 0.7770 | 0.096 | 0.166 | 0.3310 |
|  | *F*_ST_ | 0.027 | 0.009 | **0.0010** | 0.016 | 0.011 | 0.016 | 0.7640 | 0.029 | 0.003 | **0.0220** | 0.018 | 0.008 | 0.4340 | 0.022 | 0.010 | 0.2480 |

IP, all individuals pooled; M, mature trees; Sa, saplings; *A*_R_, mean allelic richness; *H*_O_, mean observed heterozygosity; *H*_E_, mean expected heterozygosity; *F*_ST_, mean pairwise *F*_ST_; *F*_IS_, inbreeding coefficient; D, discontinuous zone; C, continuous zone. Significant values (α = 0.05) given in bold type.

**Table S7** Comparison of mean genetic variability estimates between cohorts and cohorts in zones, transects, and zones within each transect of sugar maple (*Acer saccharum*) in Québec.

| Genetic  Indices | Cohorts | | | Zones | | | | | | Transects | | | | | | | | |
| --- | --- | --- | --- | --- | --- | --- | --- | --- | --- | --- | --- | --- | --- | --- | --- | --- | --- | --- |
|  | M | Sa | *P*-value | D | | | C | | | 1 | | | 2 | | | 3 | | |
|  |  |  |  | M | Sa | *P*-value | M | Sa | *P*-value | M | Sa | *P*-value | M | Sa | *P*-value | M | Sa | *P*-value |
| *A*_R_ | 5.378 | 5.275 | 0.2620 | 5.262 | 5.200 | 0.6480 | 5.484 | 5.343 | 0.2320 | 5.251 | 5.271 | 0.8840 | 5.482 | 5.382 | 0.4810 | 5.414 | 5.185 | 0.1340 |
| *H*_O_ | 0.610 | 0.591 | 0.3670 | 0.603 | 0.598 | 0.8740 | 0.616 | 0.585 | 0.2500 | 0.620 | 0.612 | 0.8020 | 0.583 | 0.551 | 0.423 | 0.622 | 0.601 | 0.1340 |
| *H*_E_ | 0.693 | 0.694 | 0.8990 | 0.689 | 0.689 | 0.9790 | 0.697 | 0.698 | 0.8560 | 0.687 | 0.692 | 0.5440 | 0.693 | 0.697 | 0.6260 | 0.699 | 0.692 | 0.5450 |
| *F*_IS_ | 0.121 | 0.148 | 0.3690 | 0.126 | 0.132 | 0.8780 | 0.116 | 0.163 | 0.2460 | 0.097 | 0.097 | 0.6830 | 0.158 | 0.210 | 0.3890 | 0.111 | 0.131 | 0.7050 |
| *F*_ST_ | 0.015 | 0.017 | 0.6650 | 0.023 | 0.027 | 0.6390 | 0.009 | 0.009 | 0.9730 | 0.021 | 0.021 | 0.5470 | 0.007 | 0.011 | 0.6120 | 0.013 | 0.016 | 0.6880 |
|  | Transects & Zones | | | | | | | | | | | | | | | | | |
|  | D | | | | | | | | | C | | | | | | | | |
|  | 1 | | | 2 | | | 3 | | | 1 | | | 2 | | | 3 | | |
|  | M | Sa | *P*-value | M | Sa | *P*-value | M | Sa | *P*-value | M | Sa | *P*-value | M | Sa | *P*-value | M | Sa | *P*-value |
| *A*_R_ | 4.951 | 5.078 | 0.5040 | 5.591 | 5.457 | 0.5360 | 5.326 | 5.130 | 0.2970 | 5.552 | 5.464 | 0.6810 | 5.400 | 5.327 | 0.6900 | 5.501 | 5.240 | 0.1950 |
| *H*_O_ | 0.599 | 0.596 | 0.9700 | 0.568 | 0.565 | 0.9670 | 0.627 | 0.620 | 0.8750 | 0.641 | 0.627 | 0.7750 | 0.593 | 0.541 | 0.3120 | 0.616 | 0.582 | 0.4900 |
| *H*_E_ | 0.676 | 0.682 | 0.6350 | 0.703 | 0.703 | 0.9970 | 0.692 | 0.685 | 0.6360 | 0.696 | 0.703 | 0.6140 | 0.686 | 0.693 | 0.5420 | 0.707 | 0.698 | 0.4860 |
| *F*_IS_ | 0.114 | 0.126 | 0.8730 | 0.193 | 0.197 | 0.9610 | 0.094 | 0.096 | 0.9810 | 0.079 | 0.109 | 0.6770 | 0.135 | 0.219 | 0.2560 | 0.129 | 0.166 | 0.6150 |
| *F*_ST_ | 0.034 | 0.029 | 0.6610 | 0.013 | 0.018 | 0.7160 | 0.019 | 0.022 | 0.7590 | 0.007 | 0.003 | 0.6900 | 0.003 | 0.008 | 0.6430 | 0.006 | 0.010 | 0.7690 |

*A*_R_, mean allelic richness; *H*_O_, mean observed heterozygosity; *H*_E_, mean expected heterozygosity; *F*_ST_, mean pairwise *F*_ST_; *F*_IS_, inbreeding coefficient; D, discontinuous zone; C, continuous zone; M, mature trees; Sa, saplings. Significant values (α = 0.05) given in bold.

**Table S8** Difference in Akaike Information Criterion (ΔAIC_C_), weights () and number of estimated parameters (*K*) for model comparisons of the relative importance of stand characteristics and distance from northern limit as predictors of *A*_R_, *H*_O_, *H*_E_ and *F*_IS_ (*n* = 414), and *F*_ST_ (*n* = 23) in Québec. For brevity, only models with a ΔAIC_C_  4.0 and the next model were shown.

| Explained variables | Model | *K* | ΔAIC_C_ | ω |
| --- | --- | --- | --- | --- |
| *A*_R_ | m_ers_PS + D_north | 5 | 0.00 | 0.19 |
|  | s_ers_PS + D_north | 5 | 0.27 | 0.16 |
|  | m_ers_PS + s_ers_PS + D_north | 6 | 0.39 | 0.15 |
|  | D_north | 4 | 0.70 | 0.13 |
|  | m_ers_d + D_north | 5 | 1.24 | 0.10 |
|  | m_ers_BA + D_north | 5 | 1.67 | 0.08 |
|  | s_ers_BA +D_north | 5 | 2.68 | 0.05 |
|  | s_ers_d + D_north | 5 | 2.68 | 0.05 |
|  | m_ers_d + s_ers_d + D_north | 6 | 2.99 | 0.04 |
|  | m_ers_BA + s_ers_BA + D_north | 6 | 3.71 | 0.03 |
|  | m_ers_BA | 4 | 7.37 | 1.00 |
| *H*_O_ | m_ers_BA + s_ers_BA | 5 | 0.00 | 0.30 |
|  | m_ers_BA + s_ers_BA + D_north | 6 | 1.75 | 0.12 |
|  | m_ers_BA | 4 | 1.86 | 0.12 |
|  | m_ers_PS + D_north | 5 | 2.50 | 0.09 |
|  | m_ers_PS + s_ers_PS + D_north | 6 | 2.99 | 0.07 |
|  | m_ers_d | 4 | 3.19 | 0.06 |
|  | m_ers_BA + D_north | 5 | 3.90 | 0.04 |
|  | m_ers_d + D_north | 5 | 4.51 | 0.03 |
| *H*_E_ | m_ers_d | 4 | 0.00 | 0.09 |
|  | m_ers_PS + D_north | 5 | 0.07 | 0.09 |
|  | m_ers_BA | 4 | 0.09 | 0.09 |
|  | D_north | 4 | 0.35 | 0.08 |
|  | s_ers_PS | 4 | 0.40 | 0.07 |
|  | m_ers_PS | 4 | 0.52 | 0.07 |
|  | s_ers_PS + D_north | 5 | 0.70 | 0.06 |
|  | s_ers_BA | 4 | 0.79 | 0.06 |
|  | m_ers_PS + s_ers_PS + D_north | 6 | 1.05 | 0.05 |
|  | s_ers_d | 4 | 1.08 | 0.05 |
|  | m_ers_BA + s_ers_BA | 5 | 1.29 | 0.05 |
|  | m_ers_d + D_north | 5 | 1.41 | 0.04 |
|  | m_ers_BA | 5 | 1.95 | 0.03 |
|  | m_ers_d + s_ers_d | 5 | 1.96 | 0.03 |
|  | s_ers_BA + D_north | 5 | 2.12 | 0.03 |
|  | m_ers_PS + s_ers_PS | 5 | 2.22 | 0.03 |
|  | s_ers_d + D_north | 5 | 2.38 | 0.03 |
|  | m_ers_BA + s_ers_BA + D_north | 6 | 3.29 | 0.02 |
|  | m_ers_d + s_ers_d + D_north | 6 | 3.46 | 0.02 |
| *F*_IS_ | m_ers_BA + s_ers_BA | 5 | 0.00 | 0.22 |
|  | m_ers_BA | 4 | 1.23 | 0.12 |
|  | m_ers_BA + s_ers_BA + D_north | 6 | 1.49 | 0.10 |
|  | m_ers_d | 4 | 2.29 | 0.07 |
|  | s_ers_BA | 4 | 2.31 | 0.07 |
|  | m_ers_PS | 4 | 2.70 | 0.06 |
|  | m_ers_PS + D_north | 5 | 3.16 | 0.04 |
|  | m_ers_BA + D_north | 5 | 3.16 | 0.04 |
|  | s_ers_PS | 4 | 3.27 | 0.04 |
|  | D_north | 4 | 3.53 | 0.04 |
|  | s_ers_d | 4 | 3.65 | 0.03 |
|  | m_ers_d + s_ers_d | 5 | 4.02 | 0.03 |
| *F*_ST_ | D_north | 3 | 0.00 | 0.22 |
|  | m_ers_d + D_north | 4 | 0.41 | 0.18 |
|  | m_ers_BA + D_north | 4 | 1.71 | 0.09 |
|  | s_ers_BA + D_north | 4 | 1.98 | 0.08 |
|  | m_ers_BA | 3 | 1.99 | 0.08 |
|  | s_ers_PS + D_north | 4 | 2.83 | 0.05 |
|  | s_ers_d + D_north | 4 | 2.93 | 0.05 |
|  | m_ers_PS + D_north | 4 | 2.96 | 0.05 |
|  | m_ers_d + s_ers_d + D_north | 5 | 3.68 | 0.03 |
|  | m_ers_d | 3 | 3.83 | 0.03 |
|  | m_ers_PS | 3 | 4.26 | 0.03 |

Explanatory variable abbreviations: m_ers_BA, mature sugar maple basal area (m^2^ ha^-1^); s_ers_BA, sugar maple sapling basal area (m^2^ ha^-1^); m_ers_d, mature sugar maple density (stems ha^-1^); s_ers_d, sugar maple sapling density (stems ha^-1^); m_ers_PS, mature sugar maple population size (stems); s_ers_PS, sugar maple sapling population size (stems); D_north, distance of each site to the northern limit (km).

**Table S9** Bottleneck results based on heterozygosity excess, Mode shift and *M*-ratio.

| Transects | Zones |  | Heterozygosity excess | | | | | | Mode  shift | *M*-ratio | *θ = 1* |  | *θ = 5* |  | *θ = 10* |  |
| --- | --- | --- | --- | --- | --- | --- | --- | --- | --- | --- | --- | --- | --- | --- | --- | --- |
|  |  | Sites | IAM | TPM | | | | SMM |  |  |  |  |  |  |  |  |
|  |  |  |  | 70% | 90% | 95% | 99% |  |  |  | *M*_c_ | *P*-value | *M*_c_ | *P*-value | *M*_c_ | *P*-value |
| 1 | Discontinuous | 1-D-A | **0.03327** | 0.81539 | 0.98288 | 0.99088 | 0.99671 | 0.99860 | N | 0.805 | 0.806 | 0.434 | 0.737 | 0.942 | 0.715 | 0.987 |
|  |  | 1-D-B | **0.01041** | 0.71008 | 0.94065 | 0.96316 | 0.99203 | 0.99552 | N | 0.792 | 0.806 | 0.342 | 0.737 | 0.345 | 0.715 | 0.577 |
|  |  | 1-D-C | **0.01518** | 0.56748 | 0.91632 | 0.95063 | 0.98658 | 0.99306 | N | 0.713 | 0.806 | **0.033** | 0.737 | 0.377 | 0.715 | 0.596 |
|  |  | 1-D-D | **0.00032** | 0.86774 | 0.99800 | 0.99987 | 0.99999 | 0.99999 | N | 0.717 | 0.806 | **0.037** | 0.737 | 0.345 | 0.715 | 0.517 |
|  | Continuous | 1-C-A | **0.02158** | 0.95929 | 0.99615 | 0.99903 | 0.99979 | 0.99992 | N | 0.768 | 0.806 | 0.196 | 0.737 | 0.734 | 0.715 | 0.902 |
|  |  | 1-C-B | **0.00200** | 0.43252 | 0.85814 | 0.92924 | 0.97586 | 0.98482 | N | 0.761 | 0.806 | 0.210 | 0.737 | 0.531 | 0.715 | 0.811 |
|  |  | 1-C-C | **0.01342** | 0.89393 | 0.99088 | 0.99968 | 0.99997 | 0.99999 | N | 0.810 | 0.806 | 0.478 | 0.737 | 0.499 | 0.715 | 0.744 |
|  |  | 1-C-D | 0.14186 | 0.90927 | 0.97842 | 0.98959 | 0.99671 | 0.99800 | N | 0.768 | 0.806 | 0.203 | 0.737 | 0.797 | 0.715 | 0.927 |
| 2 | Discontinuous | 2-D-A | **0.00021** | 0.48306 | 0.68015 | 0.72456 | 0.86774 | 0.91632 | N | 0.778 | 0.806 | 0.251 | 0.737 | 0.828 | 0.715 | 0.936 |
|  |  | 2-D-C | **0.00329** | 0.75246 | 0.98816 | 0.99763 | 0.99979 | 0.99990 | N | 0.780 | 0.806 | 0.270 | 0.737 | 0.856 | 0.715 | 0.954 |
|  |  | 2-D-D | 0.18461 | 0.94065 | 0.99398 | 0.99615 | 0.99883 | 0.99961 | N | 0.792 | 0.806 | 0.350 | 0.737 | 0.921 | 0.715 | 0.820 |
|  | Continuous | 2-C-A | **0.01041** | 0.92298 | 0.98959 | 0.99800 | 0.99860 | 0.99968 | N | 0.805 | 0.806 | 0.432 | 0.737 | 0.943 | 0.715 | 0.986 |
|  |  | 2-C-B | **0.04488** | 0.93513 | 0.99088 | 0.99615 | 0.99883 | 0.99961 | N | 0.798 | 0.806 | 0.394 | 0.737 | 0.930 | 0.715 | 0.983 |
|  |  | 2-C-C | **0.04937** | 0.95063 | 0.99480 | 0.99720 | 0.99961 | 0.99979 | N | 0.775 | 0.806 | 0.233 | 0.737 | 0.817 | 0.715 | 0.915 |
|  |  | 2-C-D | 0.10607 | 0.95063 | 0.99398 | 0.99860 | 0.99997 | 0.99998 | N | 0.776 | 0.806 | 0.239 | 0.737 | 0.627 | 0.715 | 0.932 |
| 3 | Discontinuous | 3-D-A | **0.01184** | 0.38301 | 0.56748 | 0.58414 | 0.79144 | 0.85814 | N | 0.811 | 0.806 | 0.484 | 0.737 | 0.961 | 0.715 | 0.994 |
|  |  | 3-D-B | **0.00520** | 0.35094 | 0.82673 | 0.94581 | 0.97586 | 0.97842 | N | 0.755 | 0.806 | 0.142 | 0.737 | 0.665 | 0.715 | 0.820 |
|  |  | 3-D-C | **0.00010** | 0.63314 | 0.96673 | 0.99398 | 0.99860 | 0.99903 | N | 0.756 | 0.806 | 0.148 | 0.737 | 0.675 | 0.715 | 0.834 |
|  |  | 3-D-D | **0.00013** | 0.26131 | 0.61699 | 0.76585 | 0.91632 | 0.91632 | N | 0.769 | 0.806 | 0.264 | 0.737 | 0.773 | 0.715 | 0.900 |
|  | Continuous | 3-C-A | **0.03327** | 0.94065 | 0.99615 | 0.99860 | 0.99968 | 0.99974 | N | 0.719 | 0.806 | **0.042** | 0.737 | 0.363 | 0.715 | 0.539 |
|  |  | 3-C-B | **0.02997** | 0.90927 | 0.99306 | 0.99552 | 0.99832 | 0.99860 | N | 0.813 | 0.806 | 0.483 | 0.737 | 0.961 | 0.715 | 0.990 |
|  |  | 3-C-C | **0.00385** | 0.98288 | 0.99800 | 0.99979 | 0.99987 | 0.99994 | N | 0.737 | 0.806 | 0.084 | 0.737 | 0.521 | 0.715 | 0.705 |
|  |  | 3-C-D | **0.00200** | 0.53386 | 0.75246 | 0.86774 | 0.95063 | 0.96316 | N | 0.720 | 0.806 | 0.847 | 0.737 | 0.363 | 0.715 | 0.517 |

Significant values (α = 0.05) given in bold type for heterozygosity excess.


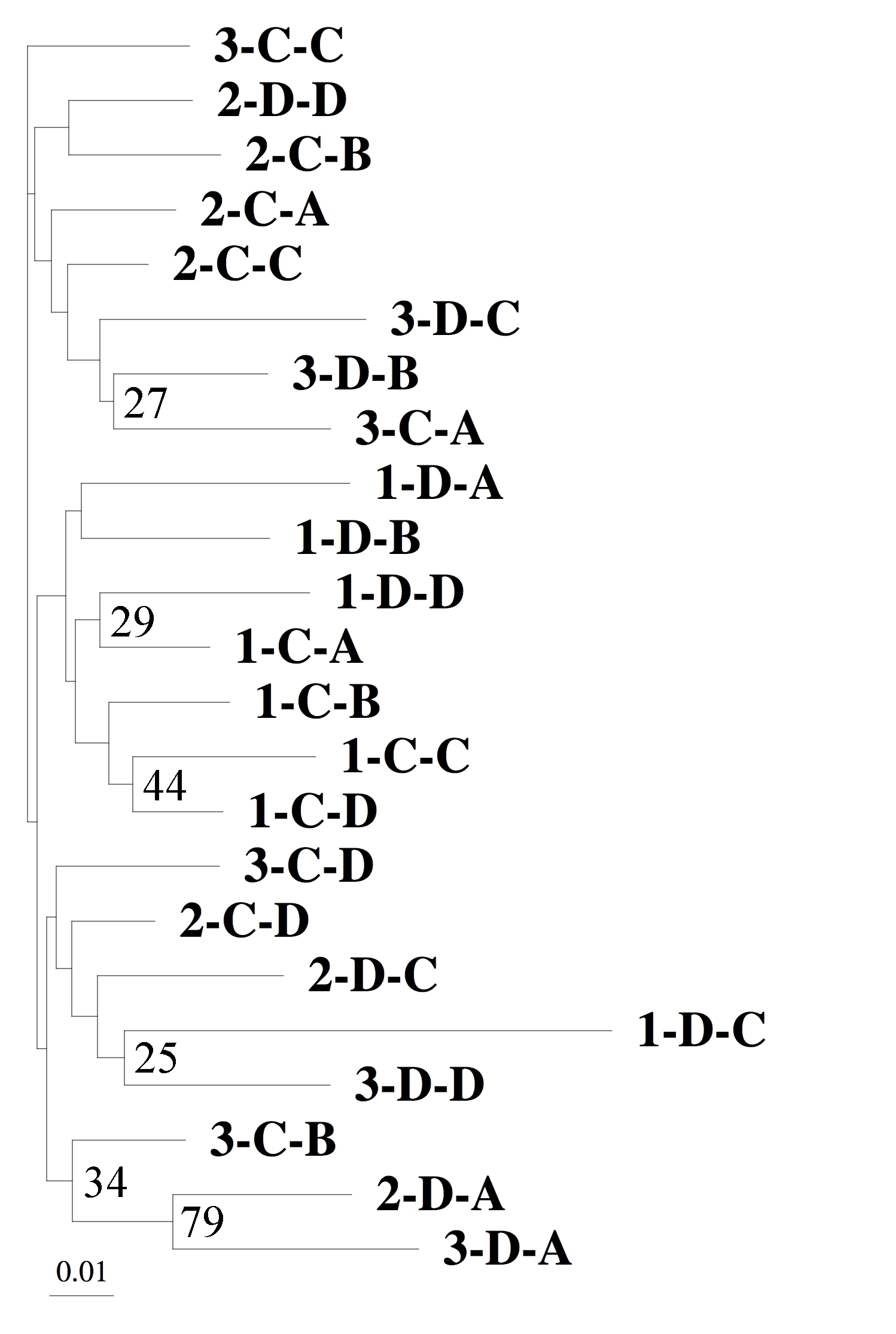

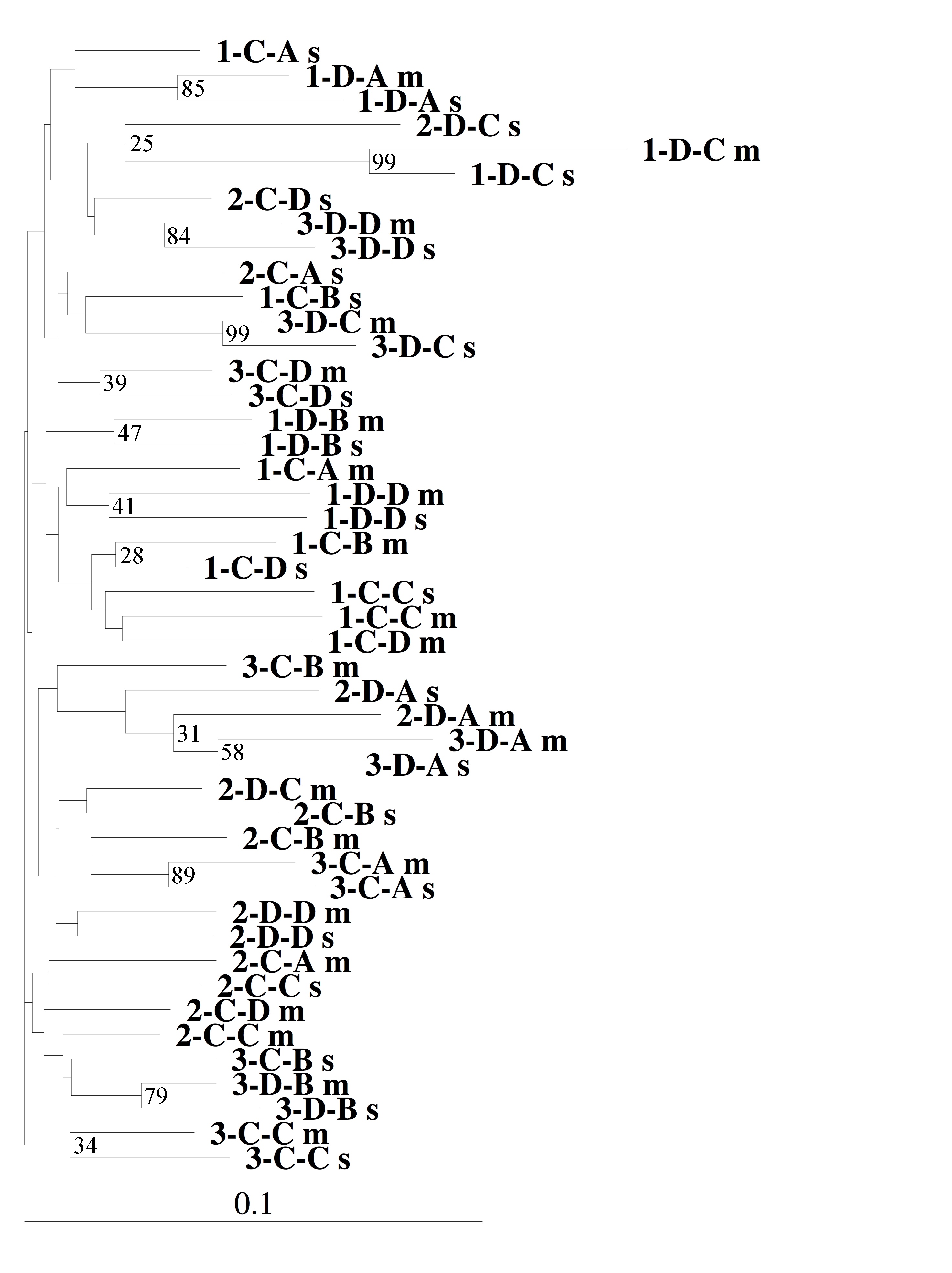


a)

b)

**Figure S1** Neighbour-joining tree based on 18 microsatellite loci (a) for the 23 sugar maple (*Acer saccharum*) sites in Québec and (b) separated into two cohorts. Genetic distances (*D*_S_) were based on Nei’s (1972) genetic distance. Numbers given in nodes indicate the support value (%) of the respective group based on 1000 bootstrapped trees. Results are shown only for branches with ≥ 25% support. Abbreviations given after site name: m, mature sugar maple and s: sugar maple sapling.


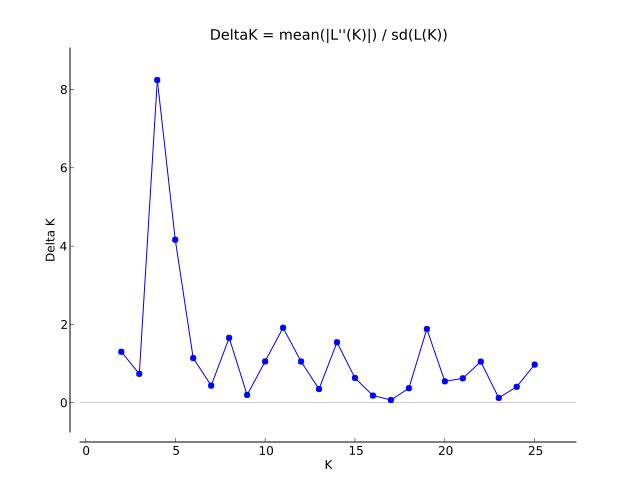


**Figure S2** Detection of the number of clusters, ∆*K* plot showing greatest support at *K* = 4, using structure for 23 sugar maple (*Acer saccharum*) sites according to Evanno *et al*. (2005).

Evanno G, Regnaut S, Goudet J (2005) Detecting the number of clusters of individuals using the software structure: a simulation study. *Molecular Ecology*, **14**, 2611–2620.

**Table S10** Results of analysis of molecular variance (AMOVA) showing the partitioning of genetic variance among transects and zones.

| Source of variation | *df* | Sum-of-  squares | Variance  components | Percentage  of variance | Phi (Φ)  statistics | *P*-values |
| --- | --- | --- | --- | --- | --- | --- |
| Original grouping: transect |  |  |  |  |  |  |
| Between transect | 2 | 132.851 | 0.108 | 1 | 0.007 | **0.000** |
| Between populations within transect | 20 | 672.927 | 0.454 | 3 | 0.028 | **0.000** |
| Within populations | 890 | 13906.340 | 15.625 | 97 | 0.035 | **0.000** |
| Total | 912 | 14712.118 | 16.187 | 100 |  |  |
| Original grouping: zone |  |  |  |  |  |  |
| Between zone | 1 | 29.1700 | 0.000 | 0 | -0.001 | 1.000 |
| Between populations within zone | 21 | 776.608 | 0.538 | 3 | 0.033 | **0.000** |
| Within populations | 890 | 13906.340 | 15.625 | 97 | 0.032 | **0.000** |
| Total | 912 | 14712.118 | 16.163 | 100 |  |  |
| Original grouping: zone in transect 1 |  |  |  |  |  |  |
| Between zone | 1 | 42.443 | 0.057 | 0 | 0.004 | **0.012** |
| Between populations within zone | 6 | 201.280 | 0.465 | 3 | 0.029 | **0.000** |
| Within populations | 303 | 4693.708 | 15.491 | 97 | 0.033 | **0.000** |
| Total | 310 | 4937.431 | 16.013 | 100 |  |  |
| Original grouping: zone in transect 2 |  |  |  |  |  |  |
| Between zone | 1 | 34.716 | 0.034 | 0 | 0.002 | *0.092* |
| Between populations within zone | 5 | 150.366 | 0.335 | 2 | 0.020 | **0.000** |
| Within populations | 275 | 4558.382 | 16.576 | 98 | 0.022 | **0.000** |
| Total | 281 | 4743.465 | 16.945 | 100 |  |  |
| Original grouping: zone in transect 3 |  |  |  |  |  |  |
| Between zone | 1 | 30.866 | 0.000 | 0 | -0.002 | 0.960 |
| Between populations within zone | 6 | 213.256 | 0.516 | 3 | 0.033 | **0.000** |
| Within populations | 312 | 4654.250 | 14.917 | 97 | 0.032 | **0.000** |
| Total | 319 | 4898.372 | 15.433 | 100 |  |  |
| structure grouping |  |  |  |  |  |  |
| Between groups | 3 | 182.386 | 0.124 | 1 | 0.008 | **0.000** |
| Between populations within group | 19 | 623.392 | 0.433 | 3 | 0.027 | **0.000** |
| Within populations | 890 | 13906.340 | 15.625 | 97 | 0.034 | **0.000** |
| Total | 912 | 14712.118 | 16.182 | 100 |  |  |

*df*, degrees-of-freedom. Significant values at α = 0.05 given in bold type and at α = 0.10 given in italics.


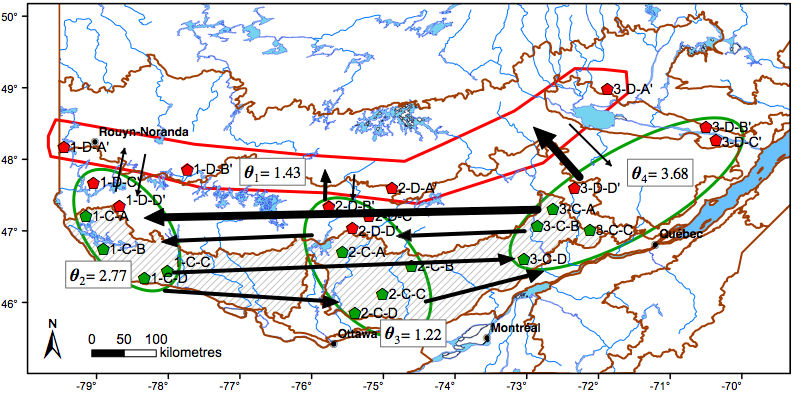


**Figure S3** Migration pattern of four groups of Québec sugar maple (*Acer saccharum*) populations, using migrate-n (Beerli 2006). The groups (red lines for the northern group and green lines for other groups) are defined using the results of structure. Mean mutation-scaled population sizes (*θ* = 4*N*_e_µ, where *N*_e_ = effective population size and *µ* = mutation rate per generation per locus) are given for each group. Arrows represent direction of migration, and the thicknesses of the arrows are proportional to mean mutation-scaled immigration rate (*M* = *m⁄µ*, where *m* = migration rate; small arrow, *M* = 6.6–8.5; medium arrow, *M* = 15.0–17.8; big arrow, *M* = 21–26). Locations of the 23 study sites (2-D-B was not included in genetics analysis) are given in red polygons for sites in discontinuous zone, and given in green polygons for sites in the continuous zone (for more details, see Graignic *et al.* 2014). Sugar maple–yellow birch (*Betula alleghaniensis*) bioclimatic domains are in grey hatching and the boundary of all bioclimatic domains are shown as thin brown lines (Saucier *et al*. 2003).

Beerli P (2006) Comparison of Bayesian and maximum-likelihood inference of population genetic parameters. *Bioinformatics* **22**:341–345.

Graignic N, Tremblay F, Bergeron Y (2014) Geographical variation in reproductive capacity of sugar maple (*Acer saccharum* Marshall) northern peripheral populations. *Journal of Biogeography* **41**, 145-157.

Saucier J-P, Grondin P, Robitaille A, Bergeron J-F (2003) Zones de végétation et domaines bioclimatiques du Québec. Ministère des Ressources naturelles, de la Faune et des Parcs (MRNFP), direction des inventaires forestiers. Publication No 2003-3015, 2 p. Available at <http://www.mrnf.gouv.qc.ca/publications/forets/connaissances/zone-vegetation-2003.pdf> (accessed 7 august 2012).

**Table S11** Comparison of genetic diversity using allozyme and microsatellite markers for old-growth, undisturbed or natural populations of tree species in northeastern North America, and *Acer* species around the world.

| Species | *A* | *A*_R_ | *H*_O_ | *H*_E_ | *F*_IS_ | *F*_ST_ / *G*_ST_ | Reference |
| --- | --- | --- | --- | --- | --- | --- | --- |
| Allozymes |  |  |  |  |  |  |  |
| Angiosperm |  |  |  |  |  |  |  |
| *Acer platanoides* | 1.88 (1.50–2.17) | — | 0.134 (0.085–0.179) | 0.133 (0.090–0.172) | -0.014 (-0.379–0.185) | — | Rusanen *et al*. (2000)* |
| *Acer platanoides* | 2.0 (1.6–2.4) | — | 0.126 (0.038–0.195) | 0.132 (0.053–0.191) | 0.066 (-0.085–0.285) | 0.099 | Rusanen *et al*. (2003) |
| *Acer pseudoplatanus* | 2.78 (2.56–3.00) | — | 0.293 (0.237–0.327) | 0.280 (0.254–0.319) | -0.032 (-0.159–0.085) | 0.019 (0.003–0.035) | Belletti *et al*. (2007) |
| *Acer macrophyllum* | 1.71 (1.5–2.2) | — | 0.118 (0.102–0.160) | 0.152 (0.102–0.189) | 0.166 (-0.086–0.332) | 0.054 | Iddrisu & Ritland (2004) |
| *Acer saccharum* | 1.95 (1.64–2.18) | — | — | 0.110 (0.098–0.132) | — | 0.033 | Perry & Knowles (1989) |
| *Acer saccharum* | 2.9 | — | 0.15 | 0.148 | — | 0.012 | Foré *et al*. (1992) |
| *Acer saccharum* | 2.03 | — | — | 0.109 | 0.073 | 0.017 | Young *et al*. (1993a)* |
| *Acer saccharum* | 1.98 (1.78–2.41) | — | — | 0.112 (0.088-0.138) | 0.042 (-0.095–0.177) | 0.033 | Young *et al*. (1993b) |
| *Acer saccharum* | 3.21 (2.50–3.75) | 2.17 (1.66–2.98) | 0.130 (0.072–0.294) | 0.133 (0.064–0.275) | 0.025 (-0.055–0.078) | 0.060 (0.060–0.114) | Baucom *et al*. (2005) |
| *Castanea dentata* | 1.69 (1.50–1.89) | — | 0.184 (0.135–0.264) | 0.151 (0.096–0.196) | -0.226 | 0.110 | Huang *et al*. (1998) |
| *Fagus grandifolia* | 2.9 (2.9–2.9) | — | 0.387 (0.382–0.392) | 0.395 (0.383–0.407) | 0.024 | 0.063 | Houston & Houston (1994) |
| *Fagus grandifolia* | 3.0 (2.78–3.33) | — | 0.163 (0.150–0.175) | 0.165 (0.150–0.179) | 0.009 | 0.030 | Houston & Houston (2000) |
| *Populus tremuloides* | 2.7 (2.1–2.9) | — | 0.125 (0.101–0.160) | 0.235 (0.207–0.270) | 0.462 (0.295–0.568) | 0.068 | Hyun *et al*. (1987) |
| *Populus tremuloides* | 2.6 (2.2–2.9) | — | 0.217 (0.197–0.242) | 0.220 (0.193–0.244) | 0.017 | 0.003 | Lund *et al*. (1992) |
| *Quercus rubra* | 2.08 (1.8–2.3) | — | — | 0.186 (0.145–0.245) | 0.100 | 0.092 | Sork *et al*. (1993) |
| Gymnosperm |  |  |  |  |  |  |  |
| *Picea glauca* | 3.03 (2.17–3.83) | 2.14 (1.86–2.37) | 0.342 (0.221–0.414) | 0.344 (0.199–0.412) | 0.002 (-0.092–0.087) | — | O'Connell *et al*. (2006) |
| *Picea rubens* | 1.47 (1.25–1.64) | — | 0.075 (0.059–0.092) | 0.079 (0.061–0.104) | 0.043 (-0.037–0.224) | 0.007 (0.003–0.011) | Hawley & DeHayes (1994) |
| *Pinus strobus* | 2.31 (2.24–2.37) | — | 0.126 (0.125–0.126) | 0.153 (0.149–0.157) | — | — | Buchert *et al*. (1997)* |
| *Pinus strobus* | 2.35 (2.23–2.50) | — | 0.215 (0.185–0.216) | 0.195 (0.181–0.216) | -0.139 (-0.273–0.407) | 0.061 | Rajora *et al*. (1998) |
| *Thuja occidentalis* | 1.6 (1.5–1.8) | — | 0.116 (0.102–0.133) | 0.129 (0.113–0.141) | 0.106 | 0.073 | Lamy *et al*. (1999) |
| Microsatellites |  |  |  |  |  |  |  |
| Angiosperm |  |  |  |  |  |  |  |
| *Acer campestre* | 6.13 (4.50–8.17) | 4.56 (3.83–5.70) | 0.473 (0.343–0.652) | 0.602 (0.509–0.699) | 0.107 (0.015–0.300) |  | Chybicki *et al*. (2014) |
| *Acer mono* | 12.63 | — | — | 0.802 | -0.008 | — | Kikuchi *et al*. (2009) |
| *Acer mono* | — | 8.37 (7.38–9.65) | — | 0.80 (0.70–0.85) | 0.27 (0.20–0.32) | — | Takayama *et al*. (2012) |
| *Acer okamotoanum* | — | 6.60 (6.11–7.41) | — | 0.72 (0.66–0.76) | 0.18 (0.03–0.24) | — | Takayama *et al*. (2012) |
| *Acer pseudoplatanus* | — | — | 0.548 (0.543–0.553) | 0.574 (0.573–0.574) | — | — | Pandey (2005) |
| *Acer pseudosieboldianum* | — | 4.60 (3.79–5.25) | 0.40 (0.32–0.46) | 0.61 (0.53–0.68) | 0.33 (0.21–0.43) | — | Takayama *et al*. (2013) |
| *Acer saccharum* | 8.2 (6.6–9.0) | 7.0 (5.8–7.6) | 0.597 (0.496–0.716) | 0.693 (0.637–0.715) | 0.138 (-0.051–0.302) | 0.016 (0.009–0.041) | Our study |
| *Acer saccharum* | 14 | — | 0.708 | 0.822 | 0.207 |  | Khodwekar *et al*. (2015) |
| *Acer saccharum* | 9.47 (9.4–9.5) | 9.0 | 0.545 (0.507–0.577) | 0.689 (0.686–693) | 0.209 (0.168–0.260) | 0.000 (-0.001–0.002) | Graignic *et al*. (2016)* |
| *Acer skutchii* | 2.1 (1.5–2.5) | — | — | 0.129 (0.054–0.247) | 0.174 (0.131–0.159) | 0.075 | Lara-Gomez *et al*. (2005) |
| *Acer takesimense* | — | 3.82 (3.59–4.23) | 0.38 (0.30–0.47) | 0.53 (0.48–0.58) | 0.28 (0.08–0.47) | — | Takayama *et al*. (2013) |
| *Quercus ellipsoidallis* | 13 | — | 0.67 (0.62–0.72) | 0.79 (0.77–0.81) | 0.145 (0.10–0.19) | 0.01 | Lind & Gailing (2013) |
| *Quercus rubra* | 14.5 (13–15) | — | 0.73 (0.70–0.75) | 0.84 (0.83–0.86) | 0.12 (0.07–0.17) | 0.02 | Lind & Gailing (2013) |
| *Populus tremuloides* | 8.83 (7.58–10.08) | — | 0.465 (0.45–0.48) | 0.67 (0.61–0.73) | 0.30 (0.21–0.39) | — | Namroud *et al*. (2005) |
| *Populus tremuloides* | — | 5.99 (3.34–6.83) | — | 0.758 (0.613–0.801) | 0.019 (-0.12–0.19) | 0.086 | Callahan *et al*. (2013) |
| *Populus tremuloides* | 7.44 (6.25–8.2) | — | 0.556 (0.478–0.704) | 0.725 (0.691–0.767) | 0.201 (-0.054–0.325) | 0.032 | Wyman *et al*. (2003) |
| Gymnosperm |  |  |  |  |  |  |  |
| *Pinus strobus* | 9.43 (9.23–9.62) | — | 0.522 (0.505–0.538) | 0.607 (0.599–0.615) | — | — | Rajora *et al*. (2000)* |
| *Pinus strobus* | — | 6.7 | 0.47 | 0.48 | 0.01 | — | Marquardt & Epperson (2004)* |
| *Thuja occidentalis* | 9.58 (7.83–11.17) | 9.21 (7.66–10.68) | 0.590 (0.505–0.640) | 0.600 (0.519–0.662) | 0.019 (-0.025–0.050) | — | Pandey & Rajora (2012a) |
| *Thuja occidentalis* | 7.3 (5.67–9.33) | 6.8 (5.16–8.51) | 0.601 (0.492–0.662) | 0.611 (0.490–0.678) | 0.013 (-0.063–0.105) | 0.078 | Pandey & Rajora (2012b) |
| *Thuja occidentalis* | 7.8 (5.0–10.0) | 5.9 (4.6–6.9) | 0.734 (0.463–0.883) | 0.773 (0.712–0.840) | 0.145 | 0.065 | Xu *et al*. (2012) |

*Study used logging or non‑natural forests; we only reported populations from old-growth and natural forests. *A*, mean number of alleles per locus; *A*_R_, mean allelic richness; *H*_O_, mean observed heterozygosity; *H*_E_, mean expected heterozygosity; *F*_IS_, inbreeding coefficient, *F*_ST_, mean pairwise *F*_ST_, *G*_ST_, mean pairwise *G*_ST_. Range values are given in parentheses.

Baucom RS, Estill JC, Cruzan MB (2005) The effect of deforestation on the genetic diversity and structure in *Acer saccharum* (Marsh): evidence for the loss and restructuring of genetic variation in a natural system. *Conservation Genetics*, **6**, 39–50.

Belletti P, Monteleone I, Ferrazzini D (2007) Genetic variability at allozyme markers in sycamore (*Acer pseudoplatanus*) populations from northwestern Italy. *Canadian Journal of Forest Research*, **37**, 395–403.

Buchert GP, Rajora OP, Hood JV, Dancik BP (1997) Effects of harvesting on genetic diversity in old-growth eastern white pine in Ontario, Canada. *Conservation Biology*, **11**, 747–758.

Callahan CM, Rowe CA, Ryel RJ, Shaw JD, Madritch MD, Mock KE (2013) Continental-scale assessment of genetic diversity and population structure in quaking aspen (*Populus tremuloides*). *Journal of Biogeography*, **40**, 1780–1791.

Chybicki IJ, Waldon-Rudzionek B, Meyza K (2014) Population at the edge: increased divergence but not inbreeding towards northern range limit in *Acer campestre*. *Tree Genetics & Genomes*, **10**, 1739–1753.

Foré SA, Hickey RJ, Guttman SI, Vankat JL (1992) Temporal differences in genetic diversity and structure of sugar maple in an old-growth forest. *Canadian Journal of Forest Research*, **22**, 1504–1509.

Graignic N, Tremblay F, Bergeron Y (2016) Genetic consequences of selection cutting on sugar maple (*Acer saccharum* Marshall). Evolutionary Applications, 9, 777–790.

Hawley GJ, DeHayes DH (1994) Genetic diversity and population structure of red spruce (*Picea rubens*). *Canadian Journal of Botany*, **72**, 1778–1786.

Houston DB, Houston DR (1994) Variation in American beech (*Fagus grandifolia* Ehrh.): isozyme analysis of genetic structure in selected stands. *Silvae Genetica*, **43**, 277–284.

Houston DB, Houston DR (2000) Allozyme genetic diversity among *Fagus grandifolia* trees resistant or susceptible to beech bark disease in natural populations. *Canadian Journal of Forest Research*, **30**, 778–789.

Huang H, Dane F, Kubisiak T (1998) Allozyme and RAPD analysis of the genetic diversity and geographic variation in wild populations of the American chestnut (*Fagaceae*). *American Journal of Botany*, **85**, 1013–1021.

Hyun JO, Rajora OP, Zsuffa L (1987) Genetic variation in trembling aspen in Ontario based on isozyme studies. *Canadian Journal of Forest Research*, **17**, 1134–1138.

Iddrisu MN, Ritland K (2004) Genetic variation, population structure, and mating system in bigleaf maple (*Acer macrophyllum* Pursh). *Canadian Journal of Botany*, **82**, 1817–1825.

Khodwekar S, Staton M, Coggeshall MV, Carlson JE, Gailing O (2015) Nuclear microsatellite markers for population genetic studies in sugar maple (*Acer saccharum* Marsh.). *Annals of Forest Research* **58**, 193–204.

Kikuchi S, Shibata M, Tanaka H, Yoshimaru H, Niiyama K (2009) Analysis of the disassortative mating pattern in a heterodichogamous plant, *Acer mono* Maxim. using microsatellite markers. *Plant Ecology*, **204**, 43–54.

Lamy S, Bouchard A, Simon J-P (1999) Genetic structure, variability, and mating system in eastern white cedar (*Thuja occidentalis*) populations of recent origin in an agricultural landscape in southern Quebec. *Canadian Journal of Forest Research*, **29**, 1383–1392.

Lara-Gomez G, Gailing O, Finkeldey R (2005) Genetic variation in isolated Mexican populations of the endemic maple *Acer skutchii* Rehd. *Allgemeine Forst und Jagdzeitung*, **176**, 97–103.

Lind J, Gailing O (2013) Genetic structure of *Quercus rubra* L. and *Quercus ellipsoidalis* E.J.Hill populations at gene-based EST-SSR and nuclear SSR markers. *Tree Genetics & Genomes*, **9**, 707–722.

Lund ST, Furnier GR, Mohn CA (1992) Isozyme variation in quaking aspen in Minnesota. *Canadian Journal of Forest Research*, **22**, 521–524.

Marquardt PE, Epperson BK (2004) Spatial and population genetic structure of microsatellites in white pine. *Molecular Ecology*, **13**, 3305–3315.

Namroud M-C, Park A, Tremblay F, Bergeron Y (2005) Clonal and spatial genetic structures of aspen (*Populus tremuloides* Michx.). *Molecular Ecology*, **14**, 2969–2980.

O'Connell LM, Mosseler A, Rajora OP (2006) Impacts of forest fragmentation on the mating system and genetic diversity of white spruce (*Picea glauca*) at the landscape level. *Heredity*, **97**, 418–426.

Pandey M (2005) Development of microsatellites in sycamore maple (*Acer pseudoplatanus* L.) and their application in population genetics. PhD thesis, Forestry Science at the Faculty of Forest Sciences and Forest Ecology, Georg-August University of Göttingen, Göttingen, Germany.

Pandey M, Rajora O (2012a) Higher fine-scale genetic structure in peripheral than in core populations of a long-lived and mixed-mating conifer - eastern white cedar (*Thuja occidentalis* L.). *BMC Evolutionary Biology*, **12**, 48.

Pandey M, Rajora OP (2012b) Genetic diversity and differentiation of core vs. peripheral populations of eastern white cedar, *Thuja occidentalis* (*Cupressaceae*). *American Journal of Botany*, **99**, 690–699.

Perry DJ, Knowles P (1989) Allozyme variation in sugar maple at the northern limit of its range in Ontario, Canada. *Canadian Journal of Forest Research*, **19**, 509–514.

Rajora OP, Deverno L, Mosseler A, Innes DJ (1998) Genetic diversity and population structure of disjunct Newfoundland and central Ontario populations of eastern white pine (*Pinus strobus*). *Canadian Journal of Botany*, **76**, 500–508.

Rajora OP, Rahman MH, Buchert GP, Dancik BP (2000) Microsatellite DNA analysis of genetic effects of harvesting in old-growth eastern white pine (*Pinus strobus*) in Ontario, Canada. *Molecular Ecology*, **9**, 339–348.

Rusanen M, Vakkari P, Blom A (2000) Evaluation of the Finnish gene-conservation strategy for Norway maple (*Acer platanoides* L.) in the light of allozyme variation. *Forest Genetics*, **7**, 155–165.

Rusanen M, Vakkari P, Blom A (2003) Genetic structure of *Acer platanoides* and *Betula pendula* in northern Europe. *Canadian Journal of Forest Research*, **33**, 1110–1115.

Sork V, Huang S, Wiener E (1993) Macrogeographic and fine-scale genetic structure in a North American oak species, *Quercus rubra* L. *Annales des sciences forestières*, **50**, 261s–270s.

Takayama K, Sun B-Y, Stuessy T (2013) Anagenetic speciation in Ullung Island, Korea: genetic diversity and structure in the island endemic species, *Acer takesimense* (*Sapindaceae*). *Journal of Plant Research*, **126**, 323–333.

Takayama K, Sun B-Y, Stuessy TF (2012) Genetic consequences of anagenetic speciation in *Acer okamotoanum* (*Sapindaceae*) on Ullung Island, Korea. *Annals of Botany*, **109**, 321–330.

Wyman J, Bruneau A, Tremblay M-F (2003) Microsatellite analysis of genetic diversity in four populations of *Populus tremuloides* in Quebec. *Canadian Journal of Botany*, **81**, 360–367.

Xu H, Tremblay F, Bergeron Y, Paul V, Chen C (2012) Genetic consequences of fragmentation in “arbor vitae,” eastern white cedar (*Thuja occidentalis* L.), toward the northern limit of its distribution range. *Ecology and Evolution*, **2**, 2506–2520.

Young A, Merriam HG, Warwick SI (1993a) The effects of forest fragmentation on genetic variation in *Acer saccharum* Marsh. (sugar maple) populations. *Heredity*, **71**, 277–289.

Young AG, Warwick SI, Merriam HG (1993b) Genetic variation and structure at three spatial scales for *Acer saccharum* (sugar maple) in Canada and the implications for conservation. *Canadian Journal of Forest Research*, **23**, 2568–2578.


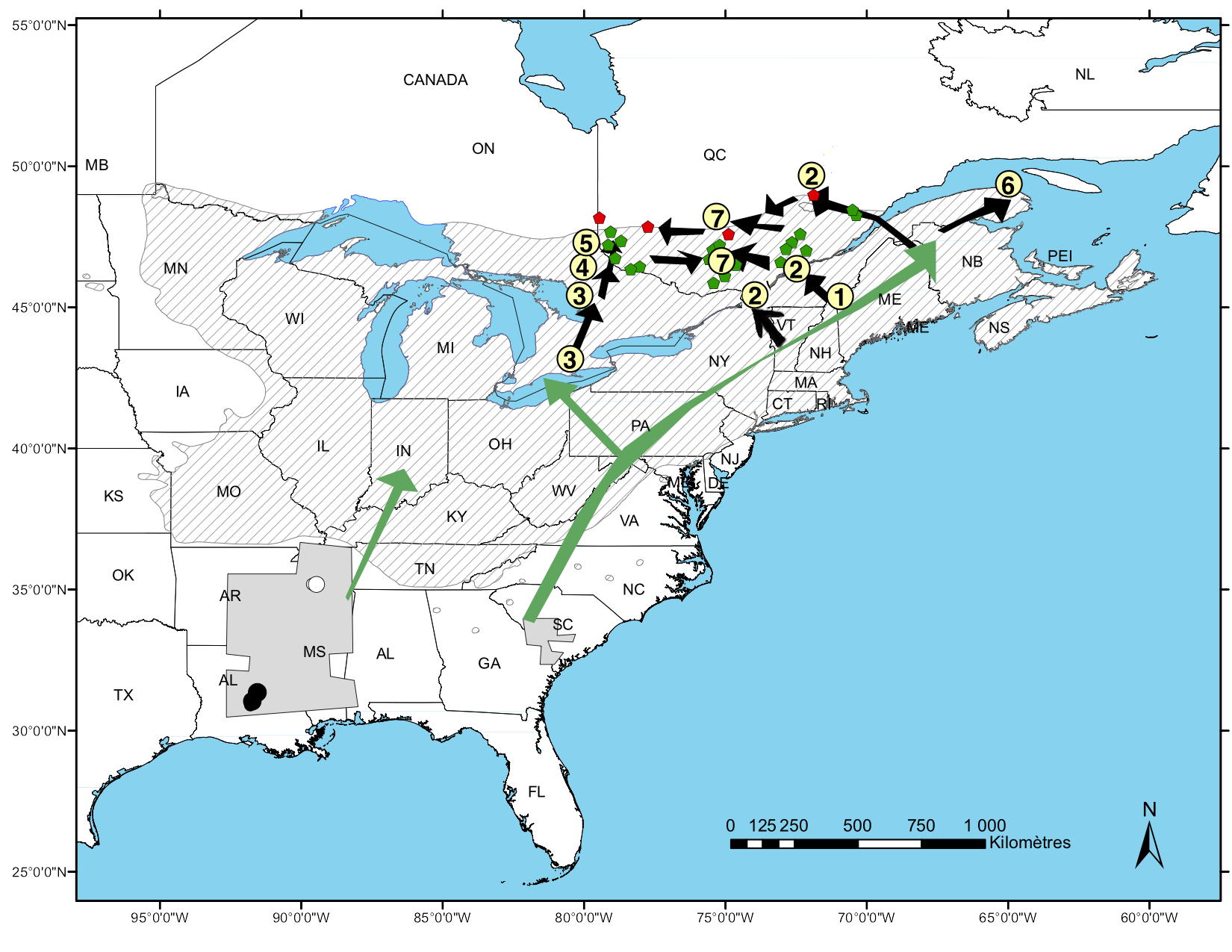


**Figure S4** Map of sugar maple migration routes. Hatched zone, sugar maple modern distribution. Grey zones, isopoll map of maple pollen in Last Glacial Maximum; black circles, presence of maple macrofossils in Last Glacial Maximum (LGM); white circle, absence of maple macrofossils in LGM (Jackson *et al*. 2000). Green arrows, hypothesized beech–maple association migration deduced from pollen (Braun 1950); black arrows, sugar maple migration deduced from our microsatellite genomic DNA study and from pollen diagrams; numbers, hypothesized chronological steps of sugar maple migration. Red polygons, northern populations group deduced from ours structure results; green polygons, our studied southern populations. Standardized two-letter state and province abbreviations were used.

Chronology of the hypothesized migration steps:

- Jackson *et al.* (2000) identified, from pollen and macrofossils data, two maple glacial refugia in the LGM (21 000 calendar years BP, cal. yr BP). West refugia is in east Louisiana and south Mississippi, and east refugia is in north Georgia and South Carolina. Interestingly, a genetic signature of these same refugia was identified for red maple (*Acer rubrum*) (McLachlan *et al.* 2005) and multiple refugia for sugar maple (Vargas-Rodriguez *et al*. 2015).
- Braun hypothesis (pollen data) suggests beech–maple associations moved northward via two routes following the ice retreat (Braun 1950). The first movement was north-east, along the Appalachians and the second was directly northward from Louisiana to North Carolina. This second movement was delayed by a warm and dry post-glacial period in Ohio and Indiana. The northern Lake States’ beech-maple forests were derived from westward expansion of the first route and not from migrations directly from the south (Braun 1950). Vargas-Rodriguez *et al*. (2015) had also shown three groups using chloroplast haplotypes.
- Earlier sugar maple migrations in Québec started approximately 9900 cal. yr BP (step 1; Lavoie & Richard 2000) in south-eastern Québec and 9600-8500 cal. yr BP (step 2; Muller & Richard 2001) west of the Lavoie & Richard (2000) study; and near Lake St-Jean (8500 cal. yr BP; Richard & Grondin 2009), and they could come from a north-westward expansion (step 2).
- Sugar maple pollen was recorded 8900 cal yr BP* in southern Ontario (step 3; Bennett 1987), just south-west of Québec 6900 cal. yr BP* (step 4; Bennett 1987) and south-west of Québec 6300 cal. yr BP* (steps 3, 4 and 5; Vincent 1973).
- Migration to Gaspésie around 6000-5500 cal. yr BP* (step 6; 5500 cal. yr BP*, Labelle & Richard 1984; and 5500 and 6000 cal. yr BP*, Marcoux & Richard 1995).
- We hypothesized that centre populations originated from an admixture of westward and eastward Québec migration (step 7). Similar routes for northernmost populations could also be drawn but with considerably greater contributions from central-eastern populations (step 7). We deduced this because very low contributions from the southwestern populations to northernmost western populations were found (Table 7; Fig. S3). In addition, western populations arrived later than did the eastern populations in Québec (Vincent 1973).

*Dates in conventional years were calibrated to calendar years by using the program CALIB 7.10 (Stuiver *et al.* 2017; http://calib.org/calib/calib.html)

Bennett KD (1987) Holocene history of forest trees in southern Ontario. *Canadian Journal of Botany*, **65**, 1792–1801.

Braun EL (1950) *Deciduous forests of eastern North America*. Afner press, New York, New York, USA. 596pp.

Jackson ST, Webb RS, Anderson KH, Overpeck JT, Webb III T, Williams JW, Hansen BCS (2000) Vegetation and environment in eastern North America during the Last Glacial Maximum. *Quaternary Science Reviews*, **19**, 489–508.

Labelle C, Richard PJH (1984) Histoire de la Végétation dans la région de Mont-Saint-Pierre, Gaspésie, Québec. *Geographie Physique et Quaternaire*, **38**, 257–274.

Lavoie M, Richard PJH (2000) Postglacial water-level changes of a small lake in southern Québec, Canada. *The Holocene*, **10**, 621–634.

Marcoux N, Richard PJH (1995) Végétation et fluctuations climatiques postglaciaires sur la côte septentrionale gaspésienne, Québec. Canadian Journal of Earth Sciences, **32**, 79–96.

McLachlan JS, Clark JS, Manos PS (2005) Molecular indicators of tree migration capacity under rapid climate change. *Ecology*, **86**, 2088–2098.

Muller SD, Richard PJH (2001) Post-glacial vegetation migration in conterminous Montréal Lowlands, southern Québec. *Journal of Biogeography* **28**, 1169-1193.

Richard PJH, Grondin P (2009) Histoire postglaciaire de la végétation. *Manuel de foresterie. Chapitre 4*, pp. 170–176. Ordre des ingénieurs forestiers du Québec, 2è édition, ouvrage collectif, éditions Multimondes, 1510 pp. Québec, Canada.

Stuiver M, Reimer P.J., Reimer RW (2017) CALIB 7.1 [WWW program] at http://calib.org, accessed 2017-2-2

Vargas-Rodriguez YL, Platt WJ, Urbatsch LE, Foltz DW (2015) Large scale patterns of genetic variation and differentiation in sugar maple from tropical Central America to temperate North America. *BMC Evolutionary Biology* **15**, 1–14.

Vincent JS (1973) Palynological study for the Little Clay Belt, northwestern Quebec. *Naturaliste Canadien*, **100**, 59–69.
